# Human and mouse monocytes display distinct signalling and cytokine profiles upon stimulation with FFAR2/FFAR3 short-chain fatty acid receptor agonists

**DOI:** 10.1038/srep34145

**Published:** 2016-09-26

**Authors:** Zhiwei Ang, Jun Zhi Er, Nguan Soon Tan, Jinhua Lu, Yih-Cherng Liou, Johannes Grosse, Jeak Ling Ding

**Affiliations:** 1 NUS graduate School for Integrative Science and Engineering, National University of Singapore, 117543 Singapore; 2 School of Biological Sciences, Nanyang Technological University, 60 Nanyang Drive, 637511, Singapore; 3 Institute of Molecular and Cell Biology, 61 Biopolis Drive, Proteos, 138673 Singapore; 4 Department of Microbiology, Yong Loo Lin School of Medicine, National University of Singapore, 117597 Singapore; 5 Department of Biological Sciences, Faculty of Science, National University of Singapore, 117543 Singapore; 6 Takeda Cambridge Limited, 418 Cambridge Science Park, Milton Road, Cambridge, CB4 0PA, UK

## Abstract

Knockout mice studies implicate the mammalian short-chain fatty acid (SCFA) receptors, FFAR2 and FFAR3– in colitis, arthritis and asthma. However, the correlation with human biology is uncertain. Here, we detected FFAR2 and FFAR3 expression in human monocytes via immunohistochemistry. Upon treatment with acetate SCFA or FFAR2- and FFAR3-specific synthetic agonists, human monocytes displayed elevated p38 phosphorylation and attenuated C5, CCL1, CCL2, GM-CSF, IL-1α, IL-1β and ICAM-1 inflammatory cytokine expression. Acetate and FFAR2 agonist treatment also repressed Akt and ERK2 signalling. Surprisingly, mouse monocytes displayed a distinct response to acetate treatment, elevating GM-CSF, IL-1α, and IL-1β cytokine expression. This effect persisted in FFAR2/3-knockout mouse monocytes and was not reproduced by synthetic agonists, suggesting a FFAR2/3 independent mechanism in mice. Collectively, we show that SCFAs act via FFAR2/3 to modulate human monocyte inflammatory responses– a pathway that is absent in mouse monocytes.

The gut microbiota has been associated with immune development and metabolic functions which include allergies, colon cancer, and inflammatory bowel disease (IBD)^1^. Some of these beneficial host-microbiome effects are mediated by short-chain fatty acids (SCFAs)^2^, which are produced in millimolar concentrations in the colonic lumen during the anaerobic fermentation of dietary fiber by saccarolytic gut bacteria^3^. The three most abundant SCFAs, namely acetate, butyrate and propionate, exert their physiological effects by activating the mammalian G protein-coupled receptors, FFAR2 (also known as GPR43 and FFA2) and FFAR3 (also known as GPR41 and FFA3), with a half maximal effective concentration (EC_50_) of ~0.5 mM^4^,^5^,^6^.

Knockout mice studies implicate FFAR2 and FFAR3 in chronic inflammatory diseases such as obesity, colitis, asthma and arthritis^7^,^8^,^9^,^10^,^11^,^12^,^13^,^14^,^15^. However, whether the receptors are protective or causative is inconsistent between studies^16^. For example, while Maslowski *et al*.^7^ and Smith *et al*.^10^ reported that FFAR2 knockout increases the severity of colitis; Sina *et al*.^8^ and Kim *et al*.^9^ conveyed the opposite. FFAR2 knockout is also demonstrated by Maslowski *et al*.^7^ to exacerbate the mouse asthma model while Trompette *et al*.^15^ reported no apparent effect. Of the two reports on FFAR3 knockout in mouse inflammation, Trompette *et al*.^15^ described exacerbated asthma while Kim *et al*.^9^ reported reduced colitis.

There is also a lack of agreement on the cell type responsible for the effect of FFAR2 and FFAR3. The function of FFAR2 in IBD has been variously associated with neutrophils^8^; gut epithelial cells^9^; or regulatory T cells^10^. Despite being highly expressed in monocytes^5^,^6^, little is known about the function of FFAR2 and FFAR3 in these cells. Monocytes may be an important target as these cells migrate to the colon mucosa and contribute to inflammation in both patients^17^ and mouse models^18^. Differentiated monocytes also form the principal source of intestinal macrophages^19^. In the colon mucosa, these cells are presumably exposed to the millimolar concentrations of SCFAs required to activate FFAR2 and FFAR3, with potential implications on monocyte-associated inflammatory disorders. Notably, the CD14^+^CD16^+^ monocyte subset is expanded and implicated in rheumatoid arthritis^20^ and IBD^21^.

Additional questions remain on the function of FFAR2 and FFAR3 in the human system as current published findings are based almost entirely on knockout mice models. Confirming the role of FFAR2 and FFAR3 in human tissues is necessary, considering the well-known limitations of using the mouse model to study human diseases^22^. For FFAR2, certain observations already point to the possibility of a difference in function among species. FFAR2 agonists induced the differentiation of mouse^23^ but not human^24^ adipocytes, while also inducing insulin secretion in mouse but not human islets^25^.

Here, we show that SCFAs act via FFAR2 and FFAR3 to modulate human monocyte inflammatory responses, a pathway that is absent in mouse monocytes. With pharmaceuticals targeting human FFAR2 already under development based on results from mice studies^26^, our findings caution that the mouse model may not replicate the full effect of such compounds on human physiology.

## Results

### Human monocytes express FFAR2 and FFAR3

**Figure 1:**
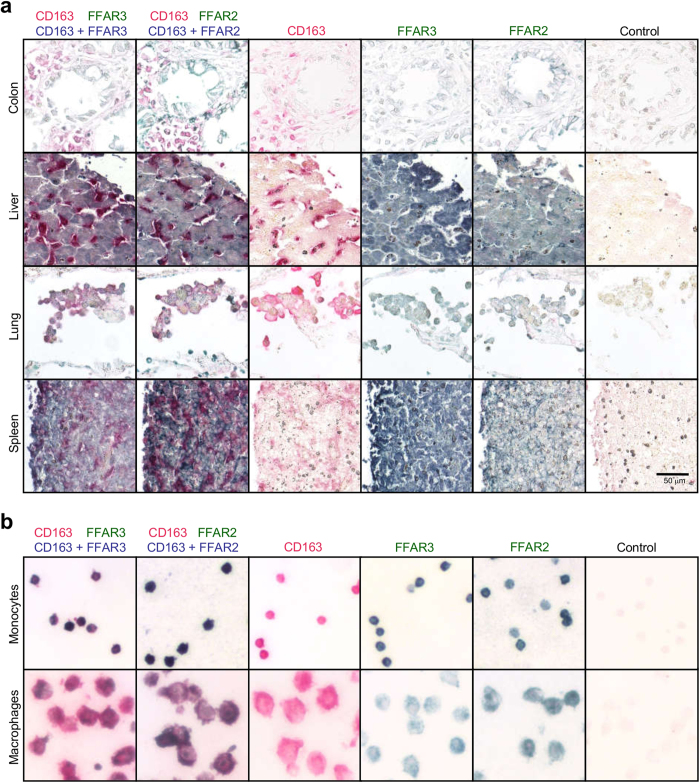
Human monocytes express FFAR2 and FFAR3 which are downregulated following differentiation into macrophages. Human tissue sections were probed via immunohistochemistry. The merged color (blue), is the result of the colocalization of antibody staining for FFAR2 or FFAR3 (both stained green) with the CD163 monocytes/macrophage marker (stained red). (**a**) Colon epithelial cells, liver hepatocytes, and the CD163+ spleen monocytes/macrophages, stained positive for FFAR2 and FFA3. Only weak FFAR2 and FFAR3 staining was observed for the CD163+ monocytes/macrophages of the colon, liver, and lung. (**b**) CD163+ peripheral blood monocytes stained positive for FFAR2 and FFAR3, and this staining was reduced when a portion of the monocyte sample was differentiated *in vitro* into macrophages. The data shown are representative of three independent experiments.

The tissue-specific expression patterns of FFAR2 and FFAR3 may provide clues into their function. Immunohistochemical staining of human tissue sections detected FFAR2 and FFAR3 expression in spleen, pancreas, liver and others [Supplementary Figure 1] [Fig. 1a]. In agreement with Karaki *et al*.^27^, we detected FFAR2 expression in colon epithelial cells, which were additionally positive for FFAR3. Since the spleen is reportedly a monocyte reservoir^28^ and based on our previous observation that *FFAR2* messenger ribonucleic acid (mRNA) is elevated in monocytes^29^, we examined FFAR3 and FFAR2 expression in relation to the monocyte and macrophage marker, CD163. CD163^+^ cells in the spleen co-stained for FFAR2 and FFAR3; while reduced FFAR2 and FFAR3 staining was observed for CD163^+^ tissue macrophages in the colon, liver and lungs [Fig. 1a]. Consistently, purified human peripheral blood monocytes stained positive for both FFAR2 and FFAR3; while reduced staining was observed in macrophages (differentiated *in vitro* from a portion of the initial monocyte sample) [Fig. 1b]. This suggests that monocytes may downregulate FFAR2 and FFAR3 expression upon differentiation into macrophages. Thus, FFAR2 and FFAR3 are expressed in human monocytes and are reduced upon differentiation into macrophages.

The lower FFAR2 expression observed in macrophages relative to monocytes is surprising, as the M-CSF cytokine which is required for monocyte maturation into macrophages, was previously found to induce monocyte *FFAR2* mRNA expression after 3 h treatment *ex vivo*^29^,^30^. We examined *FFAR2* and *FFAR3* mRNA through the entire 7-day course of monocyte differentiation into macrophages with M-CSF treatment [Supplementary Figure 2a]. Consistently, we observed an 8-fold increase in *FFAR2* mRNA at day 1 of M-CSF treatment, relative to day 0. However, this pattern was reversed from day 2 to day 7, leading to a 1-fold reduction (half the level of expression) relative to day 0. A similar trend was observed for *FFAR3* mRNA, which was attenuated by 2 folds from day 2 to day 7. This subsequent mRNA attenuation (from day 2 to day 7) was consistent with the reduced *FFAR2* and *FFAR3* protein expression observed in macrophages. Lipopolysaccharide (LPS) stimulation was previously shown to raise monocyte *FFAR2* mRNA^29^,^30^, which we also observed here [Supplementary Figure 2b]. The same treatment led to a 10-fold increase in monocyte *FFAR3* mRNA. On the other hand, treatment of macrophages with LPS led to a more modest increase of 4-fold in *FFAR2* and *FFAR3* mRNA. Taken together, *FFAR2* and *FFAR3* mRNA expression levels remain elevated in monocytes relative to macrophages, even after LPS challenge.

### FFAR2 and FFAR3 agonists reduced human monocyte inflammatory cytokine expression

**Figure 2:**
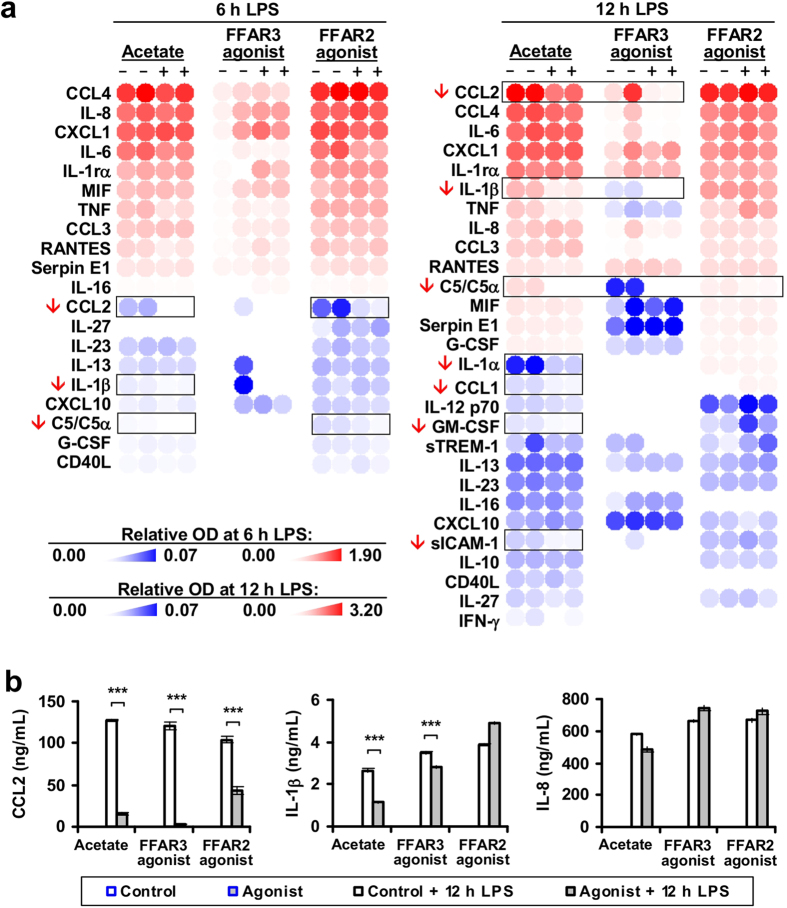
FFAR2 and FFAR3 agonists repress cytokine expression in human monocytes. Human monocytes were treated with either 5 mM acetate or 10 μM CFMB (FFAR2 agonist) or 10 μM AR420626 (FFAR3 agonist) or the respective solvent controls (NT, 0.1% v/v DMSO) for 15 min followed by 100 ng/mL LPS challenge for 6 h and 12 h. (**a**) Cytokine proteome arrays detect reduced C5, CCL1, CCL2, GM-CSF, IL-1α, IL-1β and ICAM-1 expression in acetate treated monocytes. Monocytes treated with either CFMB or AR420626 displayed reduced C5, CCL2 and IL-1β expression. Two independent cultures for each treatment condition is shown; n = 2. (**b**) Acetate- and synthetic agonist- mediated inhibition of IL-1β and CCL2 was confirmed by Enzyme-linked immunosorbent assay (ELISA). The data shown are the mean concentration from three independent cultures for each treatment condition (±SEM; n = 3) and are representative of three independent experiments.

**Figure 3:**
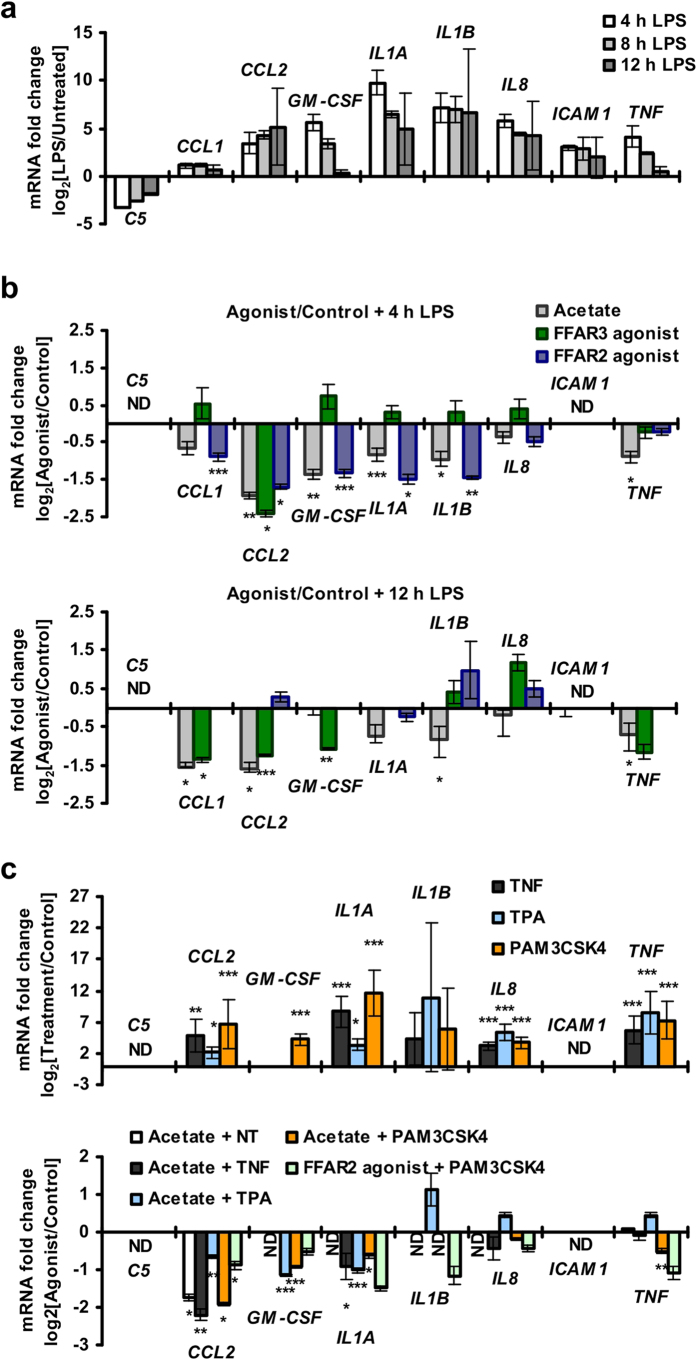
FFAR2 and FFAR3 agonists repress human monocyte cytokine mRNA in a dose dependent manner, as measured via real-time PCR. (**a**) Human monocytes upregulated inflammatory cytokines upon LPS challenge. (**b**) Human monocytes display reduced *CCL1*, *CCL2*, *CCL3*, *GM-CSF*, *IL1*α, *IL1*β and *ICAM1* upon acetate or FFAR2 and FFAR3 synthetic agonist treatment. (**c**) Acetate- and FFAR2 synthetic agonist-induced cytokine repression is observed in both naive and activated monocytes after 4 h of induction. (**a–c**) Unless otherwise indicated, human monocytes were treated with either 5 mM acetate, 10 μM CFMB (FFAR2 agonist), 10 μM AR420626 (FFAR3 agonist) or the respective solvent control for 15 min followed by a 4 h activation with inflammatory stimuli before the cytokine mRNA levels were measured by real-time analysis. Inflammatory stimuli: 100 ng/mL LPS, 10 ng/mL TNF, 200 nM 12-O-Tetradecanoylphorbol-13-acetate (TPA) and 100 ng/mL PAM3CSK4. The data shown are the means of three independent cultures for each treatment condition and is presented as the fold change of the acetate/CFMB/AR420626 treated samples relative to the respective solvent controls ± SEM; n = 3. The data shown are representative of three independent experiments. n.d: non-detectable. The two tailed Welch’s t-test was used to determine the statistical significance of the fold change (between the agonist treatment group and controls) and is annotated as: *<0.05, **<0.005, and ***<0.0005.

Since the SCFAs (acetate, propionate and butyrate) were previously found to inhibit monocyte expression of CCL2 and IL-10 cytokines *in vitro* via an unknown mechanism^31^, we investigated if SCFAs act via FFAR2 and FFAR3 to modulate these cytokines. Indeed, human peripheral blood monocyte cytokine expression was suppressed upon treatment with FFAR2- and FFAR3- specific synthetic agonists, mimicking the effect of acetate. Treatment with acetate suppressed C5, CCL1, CCL2, GM-CSF, IL-1α, IL-1β and sICAM-1 cytokine expression during LPS challenge [Fig. 2], with a corresponding reduction in mRNA [Fig. 3 and Supplementary Figure 3]. These effects were observed at the 1 to 10 mM acetate concentration used, which is within reported concentration range of acetate in the colon^3^,^32^. Specific activation of FFAR3 with the synthetic agonist, AR420626^33^, led to reduced expression of C5, CCL1, CCL2, GM-CSF and IL-1β at both the protein level [Fig. 2] and the mRNA level [Fig. 3b,c and Supplementary Figure 3]. Treatment with the FFAR2 synthetic agonist, (2S)-2-(4-chlorophenyl)-3,3-dimethyl-N-(5-phenylthiazol-2-yl)butanamide (CFMB)^34^, attenuated *CCL1*, *CCL2*, *GM-CSF*, *IL1A*, *IL1B* and *ICAM1* mRNA at 4 h post-LPS challenge [Fig. 3b,c and Supplementary Figure 3]; but not at 12 h post-LPS challenge [Fig. 3b]. Reductions at the protein level were observed for C5 and CCL2 at 6 and 12 h post-LPS challenge [Fig. 2]. The transient nature of the inhibition may be due to the purported instability of CFMB^34^. The mRNA levels may also have recovered so rapidly that there was no noticeable effect on the remaining cytokines at the protein level. This suppressive effect was not limited to LPS-activated monocytes, as acetate and CFMB treatment also reduced cytokine expression in monocytes activated by TNF, 12-O-Tetradecanoylphorbol-13-acetate (TPA) and PAM3CSK4 [Fig. 3c]. In fact, acetate reduced monocyte basal CCL2 expression in the absence of inflammatory stimuli [Fig. 3c], and during treatment with the TAK-242 inhibitor (which suppresses both ligand- dependent and -independent signalling of the LPS receptor, TLR4^35^,^36^) [Supplementary Figure 4]. On the whole, the results here suggest that FFAR2 or FFAR3 activation by either acetate or synthetic agonists, leads to reduced cytokine expression in human monocytes.

### FFAR2 and FFAR3 agonists induced human monocyte p38 phosphorylation

**Figure 4:**
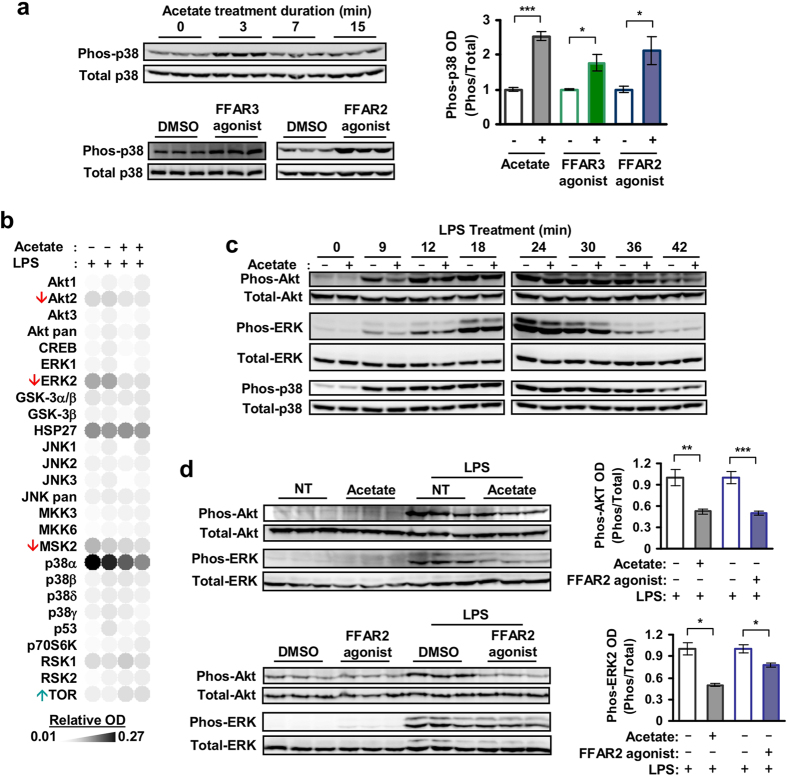
Human monocytes activate p38 in response to FFAR2 and FFAR3 agonists, and inhibit Akt and ERK2 in response to FFAR2 agonist. (**a**) Western blots show elevated phosphorylation of p38 in human monocytes at 3 min post-induction with either 5 mM acetate (top) or 10 μM FFAR2/3 synthetic agonists (bottom). Images are cropped for clarity; full-length blots are presented in Supplementary Figure 11. (**b**) Proteome array analysis of human monocytes treated with both acetate and LPS displaying reduced Akt2, ERK2 and MSK2 phosphorylation, as well as elevated TOR phosphorylation, when compared to monocytes treated with LPS alone. This experiment was performed with two independent cultures for each treatment condition. (**c**) Time course analysis showing that the acetate-mediated inhibition is more pronounced during the early stages of Akt and ERK activation by LPS in human monocytes. Images are cropped for clarity; full-length blots are presented in Supplementary Figures 12 and 13. (**d**) Western blot assays of LPS-activated human monocytes. Akt and ERK2 phosphorylation was inhibited in cells pretreated with acetate and synthetic FFAR2 agonist. Images are cropped for clarity; full-length blots are presented in Supplementary Figure 14. (**b–d**) Unless otherwise indicated, monocytes were treated for 15 min with either 5 mM acetate or 10 μM CFMB (FFAR2 agonist), followed by 100 ng/mL LPS for 8 min. (**a–d**) Each lane represents an independent culture for each treatment condition. The corresponding optical density (OD) is shown as the mean ± SEM; n = 3. The solvent controls (NT and 0.1% v/v DMSO) have been arbitrarily assigned the value of 1. The two tailed Welch’s t-test was used to determine statistical significance and is annotated as: *<0.05, **<0.005, and ***<0.0005. The data shown are representative of three independent experiments.

FFAR2 and FFAR3 may modulate cytokine expression via mitogen-activated protein kinases (MAPK) signalling^37^,^38^. Indeed, treatment of human monocytes with the FFAR2 and FFAR3 agonists (acetate, CFMB and AR420626) led to a transient increase in p38 phosphorylation that peaked at 3 min post-induction [Fig. 4a]. Only a small increase in Akt phosphorylation was observed while no elevation was detected for phosphorylated ERK and JNK [Supplementary Figure 5]. Acetate-induced activation of p38 persisted during Gi/G0 inhibition by Pertussis toxin (PT) but was abolished during Gq/11 inhibition by YM254890^39^, suggesting that Gq/11 signalling is required for the activation of p38 [Supplementary Figure 6]. Overall, these results suggest that acetate acts via FFAR2 and FFAR3 to induce p38 phosphorylation in human monocytes.

### FFAR2 agonists attenuated Akt and ERK2 phosphorylation in human monocytes

During LPS-challenge, human monocytes treated with acetate or FFAR2 agonists displayed attenuated Akt and extracellular signal-regulated kinases 2 (ERK2) phosphorylation [Fig. 4b–d]. Consistently, acetate treatment also attenuated the phosphorylation of MSK2, which is activated by ERK and p38 signalling, while the mTOR phosphorylation (a target of Akt inhibition) was enhanced [Fig. 4b]. This inhibitory effect was not observed during FFAR3 agonist treatment [data not shown] and was strongest during early activation of Akt and ERK2 by LPS [Fig. 4c]. The inhibition of Akt by acetate persisted during Gi/G0 and Gq/11 signalling inhibition by PT and YM254890 [Supplementary Figure 7a] while the inhibition of ERK2 required Gq/11 signalling and was abolished by treatment with YM254890 but not with PT [Supplementary Figure 7b]. In summary, these findings suggest that acetate acts on FFAR2 to inhibit Akt and ERK signalling.

### The human monocyte response to acetate is not mirrored by macrophages

Many of the abovementioned responses of the human monocytes to acetate, such as the induction of p38 or the attenuation of CCL2, were not observed upon *in vitro* differentiation into macrophages [Supplementary Figure 8]. Thus, human macrophage responses to acetate appear to be distinct from that of human monocytes, possibly due to reduced expression of FFAR2 and FFAR3 [Fig. 1b].

### The mouse monocyte response to acetate is distinct from human monocytes

**Figure 5:**
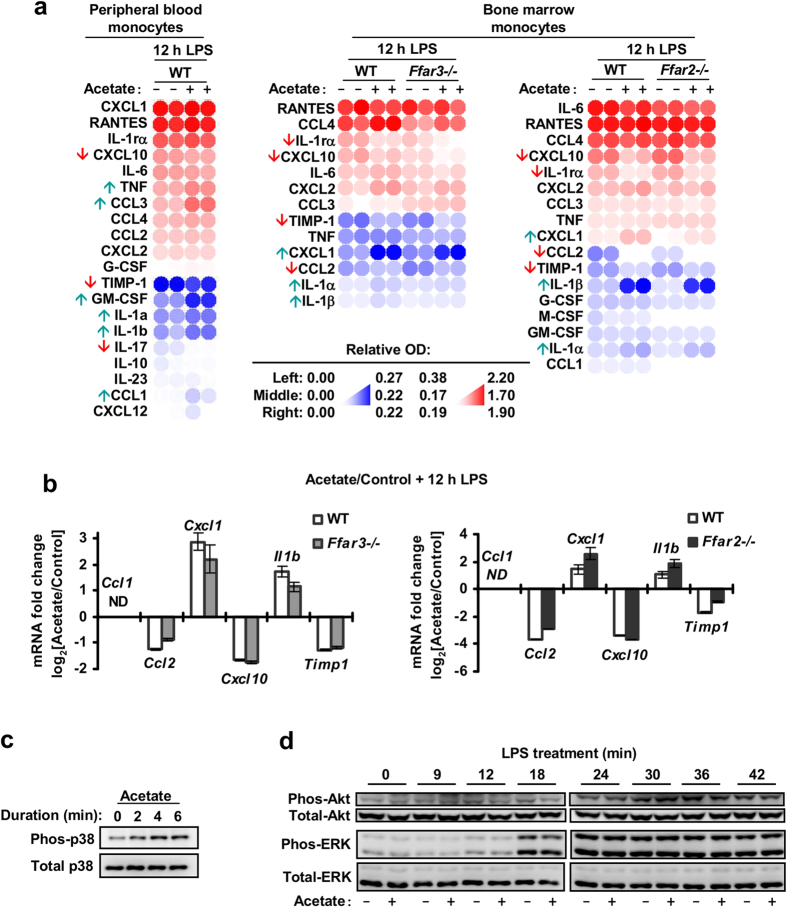
Acetate treatment of mouse monocytes led to a kinase signalling and cytokine expression profile that was distinct from human monocytes. (**a**,**b**) Mouse monocytes were treated with either 5 mM acetate or the solvent control for 15 min followed by 100 ng/mL LPS challenge for 12 h. (**a**) Cytokine expression analysis via proteome arrays. Following acetate treatment, mouse peripheral blood monocytes displayed reduced CXCL10, IL-17 and TMIP-1 expression and elevated CCL1, CCL3, GM-CSF, IL-1α, IL-1β and TNF, when compared to control monocytes treated with LPS alone. Wild type (WT), *Ffar2*^−/−^, and *Ffar3*^−/−^ mouse bone marrow monocytes, responded to acetate with reduced CXCL10, IL-1rα, TIMP-1 and CCL2, and elevated CXCL1, IL-1α and IL-1β. Two independent cultures for each treatment condition is shown; n = 2. (**b**) Real-time PCR analysis showing changes in cytokine mRNA in mouse bone marrow monocytes (WT, *Ffar2*^−/−^ and *Ffar3*^−/−^) treated with acetate. The data shown are the means of three independent cultures for each treatment condition and is presented as the fold change of the acetate treated samples relative to the solvent controls ± SEM; n = 3. n.d: non-detectable. The data shown are representative of three independent experiments. (**c**) P38 phosphorylation in mouse bone marrow monocytes during 5 mM acetate treatment. Images are cropped for clarity; full-length blots are presented in Supplementary Figure 15. (**d**) Western blot of mouse bone marrow monocytes treated with 5 mM acetate for 15 min followed by 100 ng/mL LPS challenge. No change in Akt and ERK phosphorylation levels was detected compared to controls treated with LPS alone. Images are cropped for clarity; full-length blots are presented in Supplementary Figure 16.

While FFAR2 and FFAR3 mRNA were also detected in mouse monocytes [Supplementary Figure 9a], the mouse monocyte response to acetate was distinct from human monocytes. Upon acetate treatment, mouse peripheral blood monocytes displayed reduced CXCL10, IL-17 and TMIP-1 expression and elevated CCL1, CCL3, GM-CSF, IL-1α, IL-1β and TNF, when compared to control monocytes treated with LPS alone [Fig. 5a]. Mouse bone marrow monocytes, on the other hand, responded to acetate with reduced CXCL10, IL-1rα, TIMP-1 and CCL2, and elevated CXCL1, IL-1α and IL-1β [Fig. 5a,b, and Supplementary Figure 9]. Notably, only the reduction in CCL2 was observed in both mouse bone marrow monocytes and human peripheral blood monocytes but not in mouse peripheral blood monocytes. This may be due to the larger proportion of around 70% Ly-6C^hi^ monocytes found in the bone marrow sample versus the roughly 38% Ly-6C^hi^ and 31% Ly-6C^low^ distribution found in the mouse peripheral blood monocyte sample [Supplementary Figure 10]. Ly-6C^hi^ mouse monocytes are found by gene expression arrays to more closely resemble Classical CD14^+^CD16^−^ human monocytes^40^,^41^ which make up more than 85% of the peripheral blood human monocyte sample [Supplementary Figure 10]. In mouse bone marrow monocytes, this acetate-mediated cytokine modulation persisted after FFAR2- or FFAR3-knockout [Fig. 5a] and was not reproduced by FFAR2 and FFAR3 synthetic agonists [Supplementary Figure 9d,e], suggesting a FFAR2/3 independent mechanism. In terms of kinase signalling, mouse bone marrow monocytes treated with acetate displayed elevated p38 phosphorylation [Fig. 5c]. However, acetate treatment did not reduce Akt and ERK2 phosphorylation during LPS challenge [Fig. 5d], a response that is observed in human peripheral blood monocytes [Fig. 4]. Collectively, our findings indicate that in mouse monocytes, the FFAR2 and FFAR3 agonist-mediated inhibition of Akt, ERK2 signalling and cytokine expression is absent, resulting in a response to acetate that is distinct from human monocytes.

### Heterologous expression of FFAR2 leads to p38 activation and NF-κB attenuation

**Figure 6:**
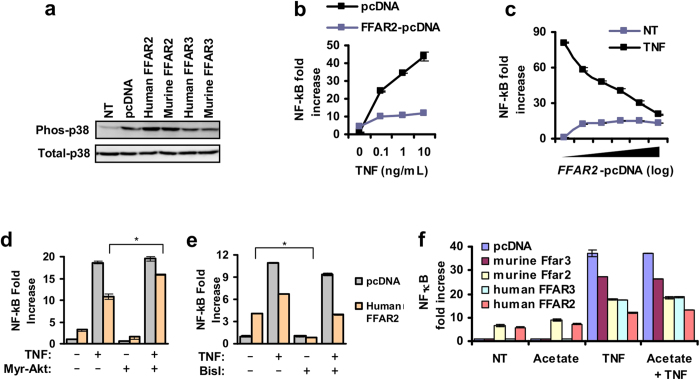
Heterologous expression of FFAR2 in A549 cells leads to the constitutive activation of p38 and attenuation of NF-κB. (**a**) Western blot shows that heterologous expression of FFAR2 leads to the constitutive phosphorylation of p38. A549 cells were transfected with FFAR2-pcDNA or FFAR3-pcDNA or the pcDNA control vector. Images are cropped for clarity; full-length blots are presented in Supplementary Figure 17. (**b**) The induction of NF-κB by TNF is reduced in A549 cells that overexpress FFAR2 versus the control cells transfected with the pcDNA control vector. (**c**) Inhibition of NF-κB activity is proportional to FFAR2 expression levels as measured by NF-κB luciferase assay. (**d**) The inhibition of NF-κB during FFAR2 expression is not affected by BisI, a PKC inhibitor. A549 cells were treated with BisI during transfection for 24 h. (**e**) The inhibition of NF-κB during FFAR2 expression is diminished when a constitutively active form of Akt (myrAkt) is present. (**f**) Among human and mouse FFAR2 and FFAR3, overexpression of human FFAR2 in A549 resulted in the strongest inhibition of NF-κB activation by TNF. (**b–f**) NF-κB luciferase reporter assays. Cells were transfected with either the FFAR2/3-pcDNA or the pcDNA control vector together with the NF-κB dual luciferase reporter plasmids. 24 h after transfection, cells were treated with 5 ng/mL TNF for 6 h before the luciferase reporters were assayed. NF-κB activity values are expressed as relative fold changes to the control cells (pcDNA control vector, no treatment). Values shown are the average of three independent cultures (n = 3) with the error bars representing mean ± SEM. The two tailed Welch’s t-test was used to determine statistical significance and is annotated as *<0.05. The data shown are representative of three independent experiments.

The function of human and mouse FFAR2 and FFAR3 was also investigated via heterologous expression in human adenocarcinoma lung alveolar basal epithelial cell line, A549 (which has low endogenous levels of FFAR2 and FFAR3 [data not shown]). Heterologous expression of FFAR2 led to the constitutive phosphorylation of p38 [Fig. 6a]. This p38 phosphorylation occurred in the absence of FFAR2 ligands, which suggests that FFAR2 is in a constitutively active state when overexpressed. Constitutive activity has been reported in some G protein–coupled receptors (GPCRs)^42^, including FFAR2 and FFAR3^5^,^43^. Lee S. U. *et al*.^44^ described inhibition of basal NF-κB activity during FFAR2 synthetic agonist treatment of Hela cells stably expressing human FFAR2^44^. Here, FFAR2 expression led to attenuated NF-κB activation by TNF [Fig. 6b,c], which is dependent on Akt^45^, as shown by the partial rescue during combined expression with myristoylated Akt (myr-Akt) [Fig. 6d]. The basal NF-κB activity (without TNF treatment), which is a pathway reportedly activated by certain GPCRs^46^, was only moderately raised by FFAR2 expression and inhibited by Bis I [Fig. 6e]. Consistent with the constitutive activation of FFAR2, the attenuation of NF-κB was proportional to the expression levels of FFAR2 [Fig. 6b] and was not increased by acetate ligand treatment [Fig. 6f]. Heterologous expression of FFAR3, albeit to a lesser extent than FFAR2, also attenuated NF-κB activity [Fig. 6f]. Collectively, these heterologous expression studies are consistent with FFAR2 mediated inhibition of Akt.

To tease out cell context dependent effects^47^, we expressed mouse FFAR2 and FFAR3 in A549 cells, obtaining the same cell context as the human receptor variants. This led to attenuated NF-κB activity [Fig. 6f], suggesting that the mouse receptor variants may also be capable of inhibitory pathway induction. The lack of FFAR2/3-mediated inhibition observed in mouse monocytes may be due to differences in signalling gene expression profiles between human and mouse monocytes^41^. Therefore, the species-specific signalling of FFAR2 and FFAR3 in monocytes is likely due to divergent monocyte function.

## Discussion

**Figure 7:**
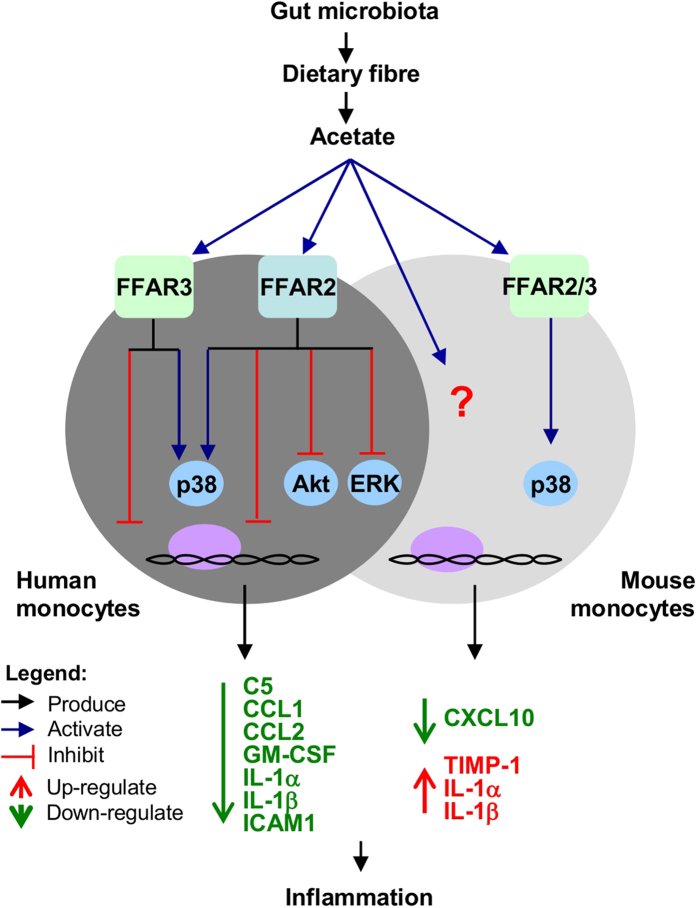
Model of the FFAR2- and FFAR3- mediated regulation of monocyte response to SCFA. The related SCFA receptors, FFAR2 and FFAR3, were found to mediate human monocyte response to SCFAs by suppressing inflammatory signalling and the resulting cytokine expression. Surprisingly, FFAR2 and FFAR3 signalling is divergent in mouse monocytes, resulting in response to acetate treatment that is distinct from human monocytes.

The role of FFAR2 and FFAR3 in colitis, arthritis and asthma is unclear as knockout mice studies have been inconsistent in whether these receptors are protective or causative^7^,^8^,^9^,^10^,^11^,^12^,^13^,^14^,^15^. Here, we sought to clarify the roles of FFAR2 and FFAR3 by examining their function in human monocytes. Interestingly, we found that FFAR2 and FFAR3 agonists attenuated Akt and ERK signalling, and cytokine expression in human but not mouse monocytes. This may account for the distinct signalling and cytokine expression profiles observed between human and mouse monocytes upon acetate treatment.This species divergence may not be restricted to monocytes since FFAR2 agonists have been found to induce insulin secretion in mouse but not human islets^25^. Many prior observations already suggest that the mouse model does not fully replicate human disease pathologies^22^. For example, while mutations in the retinoblastoma tumor suppressor gene (*RB*) in humans are causative of retinoblastoma, *Rb*^+/−^ mice show no increased incidence^48^. Our findings [summarized in Fig. 7] reveal that the mouse model, which is widely used in gut microbiota studies, does not fully replicate the distinct human response to SCFAs due to divergent FFAR2 and FFAR3 function. This may explain why current mouse models are unable to fully replicate human colitis symptoms^49^,^50^.

Many of the inflammatory cytokines attenuated by FFAR2 and FFAR3, including C5, CCL1, CCL2, GM-CSF, IL-1α, IL-1β, ICAM-1 and TNF [Figs 2 and 3], are associated with colitis. For instance, the CCL2 gene has been identified as a susceptibility loci for inflammatory bowel disease (a form of colitis)^51^ while IL-1 is essential in the initiation of colonic inflammation^52^. The potential involvement of monocyte FFAR2 and FFAR3 in human colitis, through the modulation of these cytokines, is therefore conceivable. Further investigations to determine: (i) if FFAR2 or FFAR3 signalling is altered in the monocytes of colitis patients, and (ii) whether this abnormality is involved in disease pathology– may reveal novel insights on colitis and its treatment.

Both FFAR2 and FFAR3 agonists induced p38 phosphorylation in human monocytes [Fig. 4a]. While p38 induction by FFAR2 was described for human MCF-7 breast cancer cells^53^ and mouse neutrophils^54^, here we showed that monocyte p38 can also be induced by FFAR3. p38 induction in human monocytes was GiG0 inhibitor(PT)-insensitive and Gq/11 inhibitor (YM254890)-sensitive [Fig. 4c]. This contrasts with p38 induction in mouse neutrophil which was shown to be PT-sensitive^54^, but is consistent with the PT-insensitive mechanism reported for MCF-7^54^. Finally, the functional outcome of p38 activation by FFAR2 and FFAR3 in monocytes is unclear at this juncture, and warrants future studies.

The inhibition of Akt and ERK2 phosphorylation by FFAR2 [Fig. 4b–d] is a function that has been observed in other GPCRs. Examples include the inhibition of ERK by growth factor activation of P2Y(2) GPCR in keratinocytes, which is also similarly sensitive to Gq/11 inhibitor (YM254980)^55^. FFAR2 also reportedly inhibits the induction of Akt by insulin in adipocytes in a PT-sensitive manner^14^. On the other hand, the FFAR2-mediated attenuation of monocyte Akt that we observed here was neither PT- nor YM254890- sensitive [Supplementary Figure 7a]. This suggests that distinct inhibitory pathways with distinct G protein specificities are involved. The implications of FFAR2-mediated inhibition of Akt and ERK2 in human monocytes will be an important subject of future study.

The species-specific function of FFAR2 in response to SCFA may shed light on some intriguing earlier controversies. Previously, Masui *et al*.^11^ reported that acetate treatment reduced TNF expression in both human and mouse peripheral blood mononuclear cells (PBMCs), an effect that was abolished in human monocytes by a FFAR2 antibody, but persisted in *Ffar2*^−/−^ mouse PBMCs. Our findings could possibly explain this dichotomy; that FFAR2 inhibitory activity is human monocyte-specific and therefore the inhibition of human FFAR2 activity (possibly by antibody binding) is expected to abolish the acetate-mediated cytokine inhibition. In mouse monocytes on the other hand, acetate appears to act independently of FFAR2, to modulate cytokine expression, thus *Ffar2* knockout did not abolish the inhibition.

To investigate the role of FFAR2 and FFAR3, we employed synthetic agonists and FFAR2/3 single knockouts. While FFAR2 and FFAR3 can be activated by acetate, propionate and butyrate^4^,^5^,^6^, our studies were performed with acetate to avoid known off-target effects since both butyrate and propionate reportedly inhibit histone deacetylases^56^ while butyrate also activates GPR109A^57^. The similarity in treatment outcomes between acetate and synthetic agonists (against FFAR2 and FFAR3) on human monocyte signalling and cytokine expression [Figs 2, 3 and 4], supports that FFAR2 and FFAR3 activation may account for the effect of acetate. While it is possible that both acetate and the synthetic agonists may act via the same non-specific targets to produce these effects, this scenario is unlikely as the synthetic agonists are structurally distinct from acetate and are believed to act via allosteric sites^33^,^34^. However, the synthetic agonist studies do not exclude the possibility that in addition to FFAR2 and FFAR3, SCFAs may act on other targets to induce the signalling and cytokine inhibition observed. Future studies using loss-of-function approaches involving antagonists or small interfering RNAs specific to FFAR2 and FFAR3 may elucidate the contribution of each of these receptors.

In addition to FFAR2 and FFAR3, it is likely that acetate may act on other targets as well. Notably, the FFAR2 and FFAR3 synthetic agonists do not fully mimic the effect of acetate on human monocyte cytokine expression, with the FFAR2 agonists failing to attenuate many of the cytokines at 12 h post-LPS induction [Figs 2 and 3]. While, this may be due to the purported instability of CFMB^34^, another possibility is that these later effects are mediated by other targets of acetate. These differences in cytokine regulation also suggest that pathways in addition to p38 and Akt are involved in the regulation of cytokine expression since the effect of acetate on these signalling pathways were reproduced by the synthetic agonists [Figs 4 and 5]. Consistent with the involvement of additional signalling pathway targets, we observed attenuation of cytokine expression by the FFAR3 agonist [Figs 2 and 3] despite the agonist having no noticeable effect on Akt and ERK [data not shown]. Moreover, the inhibition of Akt and Erk by acetate and the FFAR2 agonist was also transient and modest [Fig. 4]. Thus, while our findings offer a first tentative glimpse into the effect of acetate on human monocytes, it is possible that targets in addition to FFAR2 and FFAR3 are also involved, which warrants future studies.

In mouse monocytes, a distinct cytokine modulation pattern was observed during acetate treatment. This effect persisted in FFAR2 or FFAR3 knockout mice monocytes [Fig. 5a]; and was not reproduced by FFAR2/3 synthetic agonists [Supplementary Figure 9d,e]. This suggests that in mouse monocytes, the action of acetate is independent of FFAR2 and FFAR3. Interestingly, the heterologous expression of murine FFAR2 and FFAR3 in A549 cells did lead to inhibition of NF-κB, which is similar to the human homologs [Fig. 6], suggesting that the murine receptors are capable of inhibitory activity. This may be due to cell-context dependent signalling that is observed for certain GPCRs^58^. It is also possible that the synthetic agonists did not fully activate the cytokine modulation pathways downstream of murine FFAR2 and FFAR3, and that acetate is acting via the remaining receptor in mouse monocytes to fully compensate for the single receptor knockout. Further studies with FFAR2 and FFAR3 double knockout animals^59^ are required to confirm that the lack of effect in the single receptor knockouts is not due to compensation.

Due to the heterogeneity of the monocyte and macrophage population^60^, the role of FFAR2 and FFAR3 may vary from subset to subset. In fact, while it is uncertain if this was due to FFAR2/3 signalling, we observed that mouse monocytes from the bone marrow and the peripheral blood display distinct cytokine expression patterns in response to acetate [Fig. 5a]. It is possible that the FFAR2 and FFAR3 phenotype observed in the human macrophages (generated *in vitro*) used in this study may be distinct from that of other tissue resident macrophages. Notably, a population of mouse tissue resident macrophages that do not appear to be derived from circulating monocytes have been reported^61^. Likewise, since the mouse studies were performed almost entirely with bone marrow monocytes, we cannot rule out the possibility that FFAR2 and FFAR3 may have important roles in other mouse macrophage populations present in other tissues. Thus, additional studies are required to confirm the role of FFAR2 and FFAR3 in monocyte and resident tissue macrophage subsets that were not investigated in this study.

In conclusion, our study [summarized in Fig. 7], offers the first glimpse into FFAR2 and FFAR3 function in human monocytes, where these receptors modulate p38, Akt and ERK signalling [Fig. 4], and attenuate cytokine expression [Figs 2 and 3] in response to SCFA treatment. These findings hint at a potentially important role for FFAR2 and FFAR3 in mediating the interaction between the SCFA-producing gut bacteria and monocytes, with possible implications in monocyte-associated chronic inflammatory diseases^20^,^21^,^62^, in particular in IBD^17^. In mouse bone marrow monocytes, FFAR2- and FFAR3-mediated inhibition of Akt and ERK signalling and cytokine expression is not observed, accounting for a distinct response to SCFA treatment in terms of signalling and cytokine expression [Fig. 5 and Supplementary Figure 9]. This implies that despite being widely used in gut microbiota studies, the mouse model may not fully replicate the distinct human response to SCFAs due to divergent FFAR2 and FFA3 function. With the growing interest on these SCFA receptors as potential therapeutic targets^63^, our newfound insight will allow for potential differences between human and mouse monocyte responses to pharmacological compounds to be accounted for. Finally, since these preliminary observations were done with primary monocytes *ex vivo*, further investigations are required to determine the contribution of monocyte FFAR1 and FFAR3 signalling in the pathogenesis of diseases. Such studies will need to be performed with clinical samples or in mice with humanized immune systems^64^ to fully elucidate the complex interactions between the human host and its microbiome.

## Materials and Methods

### Animal work

All experimental protocols were approved by the National University of Singapore (NUS) Institutional Animal Care and Use Committee (IACUC) (Protocol Ref: 049/11, BR14/11), and were carried out in accordance with the approved guidelines. *Ffar2* and *Ffar3* knockout mice were generated as previously described^13^ and maintained in the 129/SvEv background.

### Plasmid constructs

The myristoylated human Akt1 (myr-Akt) was cloned into pcDNA3 vector or the empty pcDNA3 expression vector as control. The coding regions (CDS) of human *FFAR3* (NM_005304), human *FFAR2* (NM_005306), mouse *Ffar3* (NM_001033316), and mouse *Ffar2* (NM_001168509) were cloned into the pcDNA3.1/V5-His A (Invitrogen) expression vector.

### Primary cell isolation

Human peripheral blood monocytes were isolated from buffy coat of healthy adult donors containing citrate-phosphate-dextrose (CPD) anticoagulant (National University Hospital, Blood Donation Centre, Singapore). This work was approved by the Institutional Review Board (IRB), NUS-IRB B-14-063E, National University of Singapore (NUS). Briefly, the buffy coat was diluted four times with PBS containing 2% FBS and 1 mM EDTA, and the mononuclear fraction was obtained via density gradient centrifugation with Ficoll-Paque Premium 1.073 (GE Healthcare). From the mononuclear fraction, the monocyte population was enriched with the Human Monocyte Enrichment Kit (Stemcell). Mouse monocytes were collected from the peripheral blood and bone marrow of 8 to 14 week old 129/SvEv mice. Erythrocytes were lysed from the blood sample by diluting into an ammonium chloride solution. The monocyte fraction was enriched with the Mouse Monocyte Enrichment Kit (Stemcell).

### Macrophage generation

Enriched human peripheral blood monocytes were differentiated into macrophages by culture for 7 days in RPMI media (with 10% FBS and 1% v/v penicillin and streptomycin) supplemented with 50 ng/ml M-CSF, 37 °C, at a cell concentration of 2 × 10^6^/ml.

### Flow cytometry analysis of enriched monocyte/macrophage preparations

Monocyte/macrophage preparations were routinely analyzed by flow cytometry [Supplementary Figure 1], with mouse monoclonal antibodies from eBioscience. Enriched human peripheral blood monocyte preparations were typically more than 85% CD14^+^CD16^−^ (classical). Upon *in vitro* differentiation into human macrophages, the cell population was more than 99% CD11b^+^CD14^+^. Mouse monocyte samples were typically more than 80% CD11b^+^Ly-6G^−^. In agreement with previous reports^65^, enriched mouse peripheral blood monocyte preparations contained a mixture of around 38% Ly-6C^hi^ and 31% Ly-6C^low^ subsets, while bone marrow samples contained a higher proportion of around 70% Ly-6C^hi^ monocytes.

### Cell culture conditions

Human macrophages, peripheral blood monocytes and mouse monocytes were cultured in RPMI media (with 10% FBS and 1% v/v penicillin and streptomycin) at 37 °C for 2 h before being used in assays. The A549 adenocarcinomic human lung alveolar basal epithelial cells were routinely cultured in RPMI media (with 10% FBS and 1% v/v penicillin and streptomycin) at 37 °C.

### Luciferase reporter assays

For A549 cells, luciferase assays were performed in 24-well plates. Transfections were performed at ~80% confluency using the TurboFect Transfection Reagent (Thermo Scientific), and according to the manufacturer’s protocol. A combination of 0.8 μg expression/control vector + 0.2 μg pNFκB_Luc (Stratagene) + 0.02 μg pRL-CMBV (Promega) was used. For experiments involving the exogenous expression of multiple proteins, an equal mass of each expression vector was used to make up 0.8 μg. Following ligand induction, the luciferase activities were measured with the Dual-Luciferase® Reporter Assay kit (Promega).

### Cell treatments

Unless otherwise stated, cells were treated with the final concentration of 10 μM CFMB [(2S)-2-(4-chlorophenyl)-3,3-dimethyl-N-(5-phenylthiazol-2-yl)butanamide] (Calbiochem) (ChemSpider ID: 24656891) (EC50 of ~0.7 μM)^34^, 10 μM AR420626 [N-(2,5-Dichlorophenyl)-4-(furan-2-yl)-2-methyl-5-oxo-1,4,5,6,7,8-hexahydro-quinoline-3-carboxamide] (Glixx Laboratories) (EC50 of ~0.7 μM)^33^, 100 ng/mL *Escherichia coli* 055:B5 LPS (Sigma), 10 ng/mL TNF (Gibco) and 200 nM 12-O-Tetradecanoylphorbol-13-acetate (TPA) (Sigma), 100 ng/mL PAM3CSK4 (Invivogen). 500 ng/mL of Pertussis Toxin (PT) (Invitrogen), 10 μM YM254890^39^, 2 μM Wortmannin (Sigma), 10 μM U0126 (Cell Signalling) and 2 μM TAK-242 (Cayman Chemical)^35^,^36^.

### Immunohistochemistry

This work was approved by the Institutional Review Board (IRB), NUS-IRB B-15-155E, National University of Singapore (NUS). The human normal tissue microarrays, colon and lung sections, were obtained from Biomax.us. Human monocytes and macrophages were embedded in 1% agarose and fixed overnight with 10% neutral buffered formalin solution followed by embedding with paraffin with tissue processor (Leica). Following antigen retrieval (0.01 M Citrate buffer pH 6, 20 min at 99 °C), tissue sections were stained at 4 °C overnight with primary antibodies against CD163 (Thermo Scientific, MA5-11458), FFAR3 (Atlas Antibodies, HPA044681, Lot R40941) and FFAR2 (Santa Cruz Biotechnology, sc-32906). Secondary antibody incubation and chromomeric substrate development was then performed with the DS-MR-Hu C2 Kit (Polink).

### Proteome array and Western blot analysis of MAPKs and cytokines

The Human Phospho-MAPK Array Kit (R&D Sytems, ARY002B), Mouse Cytokine Antibody Array (R&D Systems, ARY006) and Human Cytokine Array Kit (R&D Systems, ARY005) were used. The array chemiluminescent signals were captured with ImageQuant™ LAS 4000 mini (GE Healthcare). Densitometric analysis was performed using the ImageQuant TL Software (GE Healthcare). Optical densities were standardized to the mean positive control (reference spots 1 to 3) readings and displayed as a heat map generated using the matrix2png program^66^.

Total cell lysates were resolved on a denaturing SDS PAGE gel (10%) and transferred onto Immuno-Blot PVDF membranes (Biorad). These were then probed with antibodies against XBP1 (Abcam, ab37152), β-actin (Sigma, a2066), phospho-p38 (Thr180/Tyr182) (Cell Signalling, 4511), p38 (Cell Signalling, 8690), phospho-Akt (Ser437) (Cell Signalling, 4060), Akt (Cell Signalling, Cat. #9272), phospho-ERK1/2 (Thr202/Tyr204) (Cell Signalling, 4370), ERK1/2 (Cell Signalling, Cat. #9107), phospho-SAPK/JNK (Thr183/Tyr185)(Cell Signalling, #9255) or SAPK/JNK Antibody (Cell Signalling, Cat. #9252). These primary antibodies were then probed with the respective HRP-conjugated secondary antibodies: Goat anti-Rabbit (Dako, P00448) or Goat Anti-Mouse (Dako, P0447). Western blot chemiluminescent signals were captured with an ImageQuant™ LAS 4000 mini (GE Healthcare). Densitometric analysis was performed on biological triplicates using the ImageQuant TL Software (GE Healthcare). These PVDF blots were then stripped before further reprobing.

### Real-time quantitative PCR analysis of cytokine mRNA

RNA was extracted using the Trizol Reagent (Ambion). The purified RNA was reverse transcribed with the SuperScript® III First-Strand Synthesis System (Invitrogen) and analyzed by real-time qPCR using the GoTaq® qPCR Master Mix (Promega) and a LightCycler® 480 (Roche). Primers, all with an annealing temperature of 60 °C, are listed in Table 1. With *RPL27* as the reference gene, the mean fold change of the treatment condition relative to the reference condition was calculated using the Relative Expression Software Tool (REST MCS © - version 2)^67^,^68^.

### Statistical analysis

Data are presented as means ± SEM of triplicate conditions/samples tested and are representative of at least 3 independent experiments. Differences between averages were analyzed by the two-tailed Welch’s unequal variance t-test. Significance was set at a P-value of <0.05. *P < 0.05; **P < 0.005; *P < 0.0005.

## Additional Information

**How to cite this article**: Ang, Z. *et al*. Human and mouse monocytes display distinct signalling and cytokine profiles upon stimulation with FFAR2/FFAR3 short-chain fatty acid receptor agonists. *Sci. Rep*. **6**, 34145; doi: 10.1038/srep34145 (2016).

## Supplementary Material

Supplementary Information

## Figures and Tables

**Table 1:** List of PCR primers.

Human mRNA	Mouse mRNA
RPL27_F;ATCGCCAAGAGATCAAAGATAA	Rpl27_F;AACTACAACCACCTCATGCCC
RPL27_R;TCTGAAGACATCCTTATTGACG	Rpl27_R;TCCCTGTCTTGTATCGCTCCT
C5_F;AGCCAGCCAAAAGAGAAACTGTC	C5_F;CTGCTTGAAAACACCCTGCC
C5_R;ATGCGGTTCCAGTTGTTGAAAAG	C5_R;AGCTGTCTGGACGTTTGAGG
CCL1_F;GGAAGATGTGGACAGCAAGAGC	Ccl1_F;TTCCCCTGAAGTTTATCCAGTGTT
CCL1_R;TGTAGGGCTGGTAGTTTCGG	Ccl1_R;TGAACCCACGTTTTGTTAGTTGAG
CCL2_F;GATCTCAGTGCAGAGGCTCG	Ccl2_F;CCCAATGAGTAGGCTGGAGA
CCL2_R;TGCTTGTCCAGGTGGTCCAT	Ccl2_R;TTGGTTCCGATCCAGGTTTTTAA
FFAR2_F;GCCTGGTGCTCTTCTTCATC	Cxcl1_F;TGCACCCAAACCGAAGTCAT
FFAR2_R;AGGTGGGACACGTTGTAAGG	Cxcl1_R;TTGTCAGAAGCCAGCGTTCAC
FFAR3_F;CACCATCTATCTCACCGCCC	Cxcl2_F;TCCAGAGCTTGAGTGTGACG
FFAR3_R;TATGACGTAGACCACGCTGC	Cxcl2_R;TTCAGGGTCAAGGCAAACTT
GM-CSF_F;CACTGCTGCTGAGATGAATGAAA	Cxcl10_F;GACGGTCCGCTGCAACTG
GM-CSF_R;GTCTGTAGGCAGGTCGGCTC	Cxcl10_R;GCTTCCCTATGGCCCTCATT
ICAM1_F;GGCTGGAGCTGTTTGAGAAC	Ffar2_F;TTCCCATGGCAGTCACCATC
ICAM1_R;ACTGTGGGGTTCAACCTCTG	Ffar2_R;TGTAGGGTCCAAAGCACACC
IL1A_F;GAATGACGCCCTCAATCAAAGT	Ffar3_F;TCCTGCCGTTTCGCATGGTGG
IL1A_R;TCATCTTGGGCAGTCACATACA	Ffar3_R;ACCGCCGTCAGGAAGAGGGAG
IL1B_F;AAGCTGAGGAAGATGCTG	GM-CSF_F;TGTGGTCTACAGCCTCTCAGCAC
IL1B_R;ATCTACACTCTCCAGCTG	GM-CSF_R;CAAAGGGGATATCAGTCAGAAAGGT
IL8_F;TGTGAAGGTGCAGTTTTGCCAAGG	Icam1_F;CAATTTCTCATGCCGCACAG
IL8_R;GTTGGCGCAGTGTGGTCCACTC	Icam1_R;AGCTGGAAGATCGAAAGTCCG
TNF_F;CCCCAGAGGGAAGAGTTCCCCA	Il1A_F;TTGGTTAAATGACCTGCAACA
TNF_R;GGCGGTTCAGCCACTGGAG	Il1A_R;GAGCGCTCACGAACAGTTG
	Il1B_F;TGTAATGAAAGACGGCACACC
	Il1B_R;TCTTCTTTGGGTATTGCTTGG
	Il1ra_F;CTTTACCTTCATCCGCTCTGAGA
	Il1ra_R;TCTAGTGTTGTGCAGAGGAACCA
	Timp1_F;TGTGGGAAATGCCGCAGATA
	Timp1_R;TTCACTGCGGTTCTGGGACT
	Tnf_F;CTGTAGCCCACGTCGTAGC
	Tnf_R;TTGAGATCCATGCCGTTG
