# Supplementary material for: Human and mouse monocytes display distinct signalling and cytokine profiles upon stimulation with FFAR2/FFAR3 short-chain fatty acid receptor agonists
Source: Sci Rep. 2016 Sep 26;6:34145. doi: 10.1038/srep34145 (PMC5036191; doi:10.1038/srep34145)
Supplement: Supplementary Information [file srep34145-s1.pdf]

Human and mouse monocytes display distinct signaling and cytokine profiles upon stimulation with FFAR2/FFAR3 short-chain fatty acid receptor agonists

Zhiwei Ang[1], Jun Zhi Er[1], Nguan Soon Tan[2,3], Jinhua Lu[4], Yih-Cherng Liou[5], Johannes Grosse[6] and
Jeak Ling Ding[5,*]

[1] NUS graduate School for Integrative Science and Engineering, National University of Singapore, Singapore, 117543
[2] School of Biological Sciences, Nanyang Technological University, 60 Nanyang Drive, Singapore 637511
[3] Institute of Molecular and Cell Biology, 61 Biopolis Drive, Proteos, Singapore 138673.
[4] Department of Microbiology, Yong Loo Lin School of Medicine, National University of Singapore, Singapore, 117597
[5] Department of Biological Sciences, Faculty of Science, National University of Singapore, Singapore, 117543
[6] Takeda Cambridge Limited, 418 Cambridge Science Park, Milton Road, Cambridge, CB4 0PA, UK

[*] Correspondence:
Jeak Ling Ding; Email: dbsdjl@nus.edu.sg; Tel: 65-6516 2776; Fax: 65-6779 2486.

Supplementary Figures

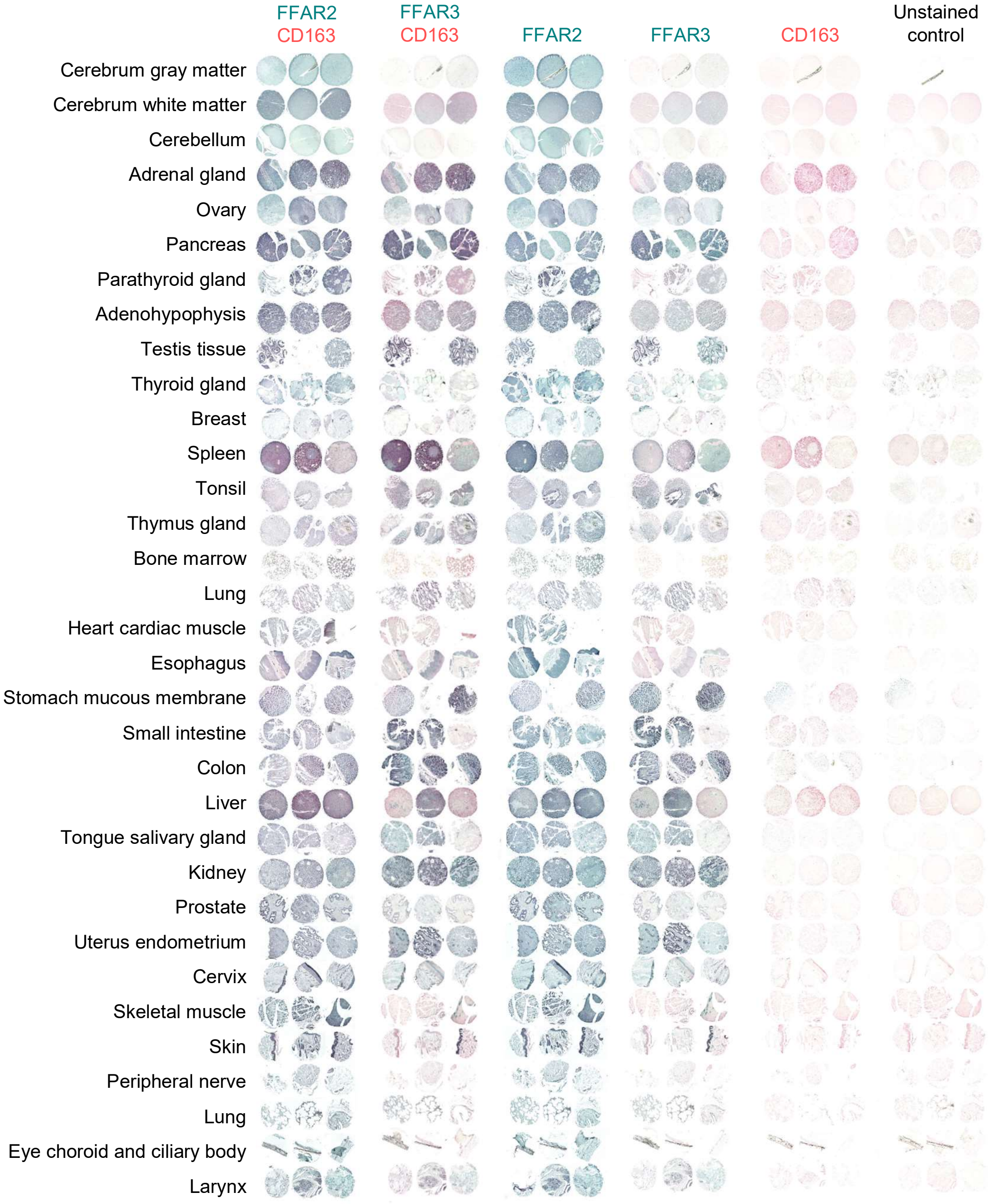

Supplementary Figure 1 | FFAR2 and FFAR3 expression is elevated in the pancreas, spleen, intestines, liver and kidney. Immunohistochemical staining of a human tissue array containing 32 tissue types each, from 3 donors (Biomax Inc, USA). The merged color (blue), is the result of the co-localization of FFAR2 or FFAR3 (both stained green) with the CD163 monocytes/macrophage marker (stained red).

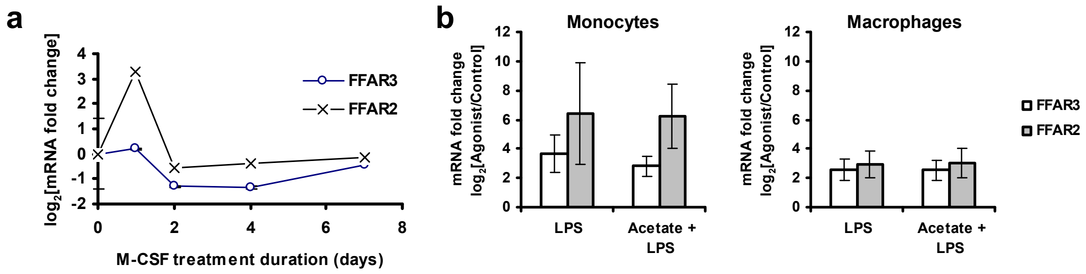

Supplementary Figure 2 | Human monocytes display elevated FFAR2 and FFAR3 mRNA expression relative to macrophages. (a) Human peripheral blood monocytes were differentiated into macrophages by culturing with 50 ng/ml M-CSF for 7 days. At the indicated times, *FFAR2* and *FFAR3* mRNA levels were measured by real-time PCR analysis. **(b)** FFAR2 and FFAR3 mRNA levels in human monocytes and macrophages after 4 h activation with 100 ng/mL LPS. **(a - b)** The data shown are the means of three independent cultures for each treatment condition and is presented as the fold change of the treated samples relative to the respective solvent/day 0 controls ± SEM; n = 3. The data shown are representative of three independent experiments. n.d: non-detectable.

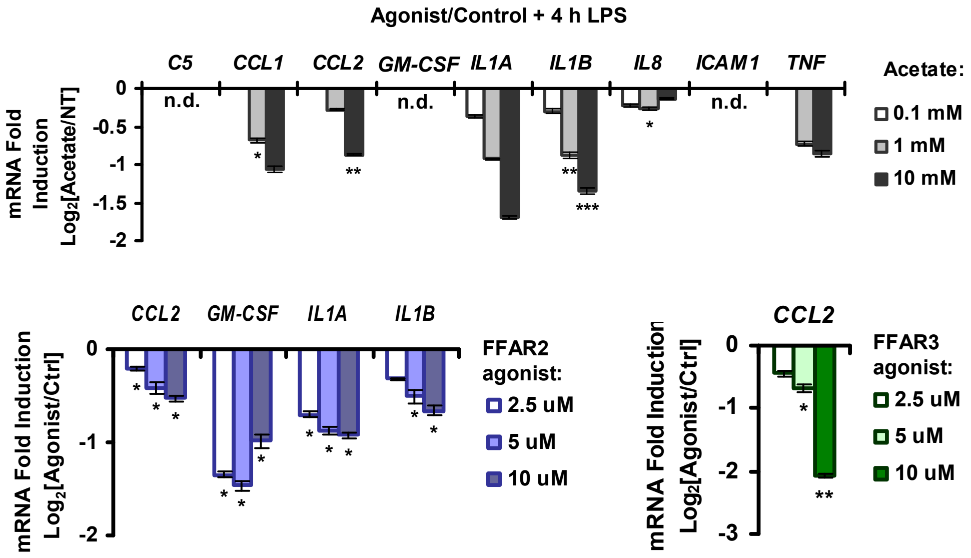

Supplementary Figure 3 | Acetate- and FFAR2/3 synthetic agonist-induced cytokine inhibition is dose-responsive. Human monocytes were treated with acetate, CFMB (FFAR2 agonist), AR420626 (FFAR3 agonist) or the respective solvent control for 15 min followed by a 4 h activation with 100 ng/mL LPS before the cytokine mRNA levels were measured by real-time PCR analysis. The data shown are the means of three independent cultures for each treatment condition and is presented as the fold change of the acetate/CFMB/AR420626 treated samples relative to the respective solvent controls ± SEM; n = 3. The data shown are representative of three independent experiments. n.d: non-detectable. The two tailed Welch's t-test was used to determine the statistical significance of the fold change (between the agonist treatment group and controls) and is annotated as: * < 0.05, ** < 0.005, and *** < 0.0005.

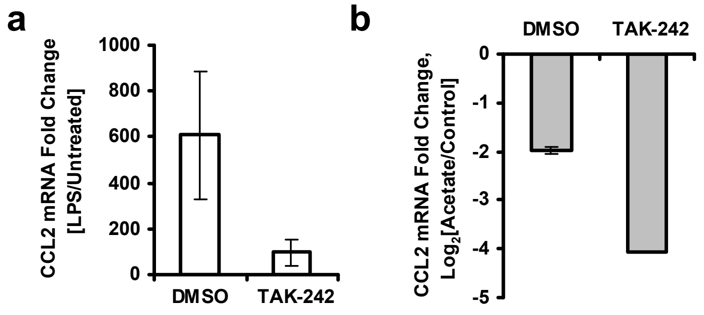

Supplementary Figure 4 | Acetate treatment suppresses basal expression of CCL2 in human monocytes. Human monocytes were pretreated for 1 h with 2uM TAK-242 or DMSO solvent control, followed by treatment with 10 mM acetate or 100 ng/mL LPS for 4 h. **(a)** Monocytes pretreated with TAK-242 display attenuated CCL2 expression upon LPS induction. **(b)** Monocytes pretreated with TAK-242 continue to display attenuated basal CCL2 expression.

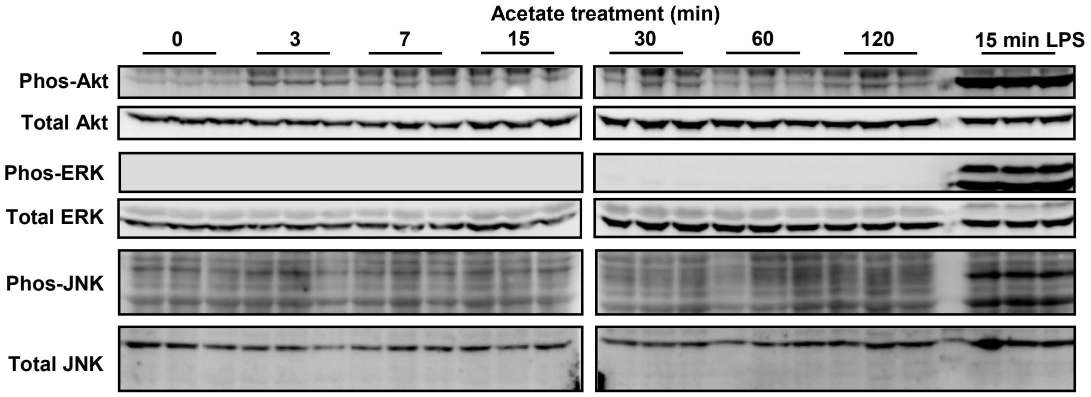

Supplementary Figure 5 | Human monocyte Akt, ERK and JNK phosphorylation levels during acetate treatment. Human monocytes were treated with 5 mM acetate. Akt, ERK and JNK phosphorylation levels were measured via western blots at the indicated time-points. Each lane represents an independent culture for each treatment condition. The data shown are representative of three independent experiments.

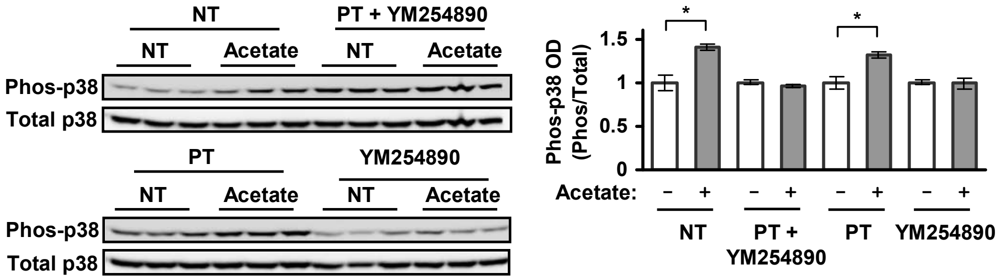

Supplementary Figure 6 | Acetate activates human monocyte p38 in a Gq/11 dependent manner. Phosphorylation of p38 by 5 mM acetate is abolished in human monocytes treated with YM254890 (Gq/11 inhibitor) alone or a combination of YM254890 and Pertussis toxin (PT, GiG0 inhibitor), but is retained in the solvent control (NT) monocytes and monocytes treated with PT alone. Cells were pretreated with PT for 2 h and/or YM254890 for 30 min before acetate induction. Each lane represents an independent culture for each treatment condition. The corresponding optical density (OD) is shown as the mean ± SEM; n = 3. The solvent controls (NT and 0.1% v/v DMSO) have been arbitrarily assigned the value of 1. The two tailed Welch's t-test was used to determine statistical significance and is annotated as: * < 0.05 and *** < 0.0005. The data shown are representative of three independent experiments.

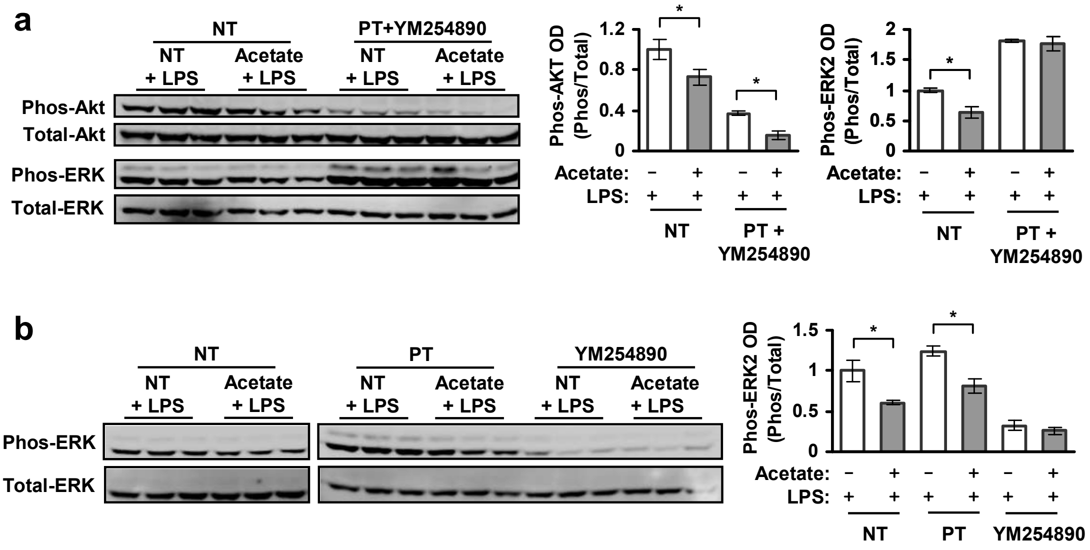

Supplementary Figure 7 | Acetate inhibits ERK2 in a Gq/11 dependent manner. (a) Combined treatment with PT and YM254890 abolished ERK2 inhibition but does not affect Akt inhibition. YM254890 treatment alone abolished ERK2 inhibition while PT has no effect. Monocytes were pretreated with PT for 2 h and/or YM254890 for 30 min. Monocytes were then treated for 15 min with 5 mM acetate, followed by 100 ng/mL LPS for 8 min. Each lane represents an independent culture for each treatment condition. The corresponding optical density (OD) is shown as the mean ± SEM; n = 3. The solvent controls (NT and 0.1% v/v DMSO) have been arbitrarily assigned the value of 1. The two tailed Welch's t-test was used to determine statistical significance and is annotated as: * < 0.05, ** < 0.005, and *** < 0.0005. The data shown are representative of three independent experiments.

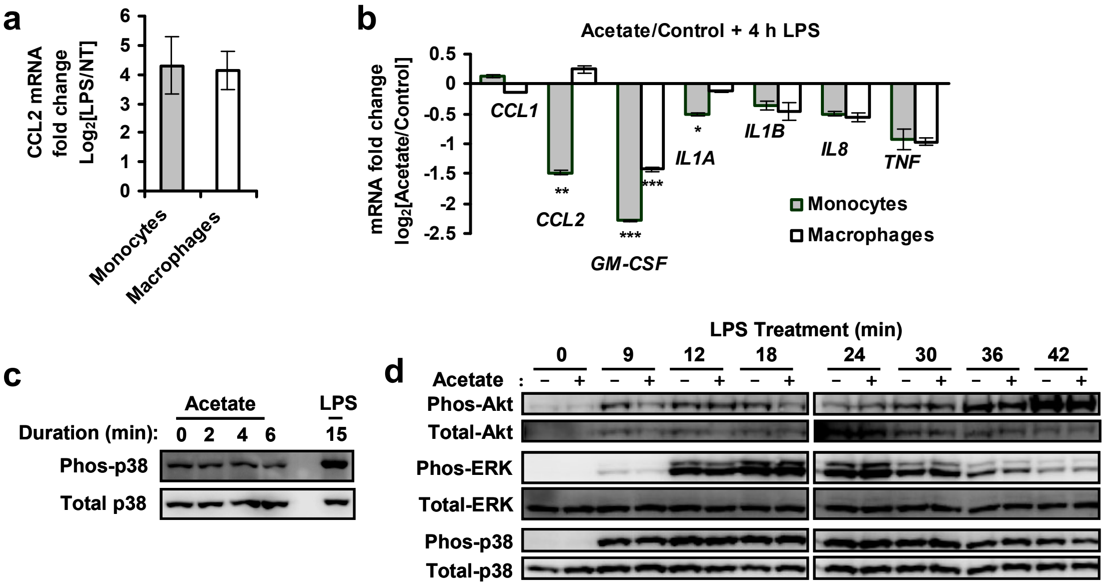

Supplementary Figure 8 | Human monocytes, and not macrophages, display attenuated CCL2 expression upon acetate treatment. (a – b) Human monocytes/macrophages were treated with 5 mM acetate or a solvent control (water) for 15 min followed by 100 ng/mL LPS. Real-time PCR analysis of cytokine mRNA. Acetate treatment failed to attenuate CCL2 expression in macrophages. Monocytes from the same donor prior to differentiation are shown as controls. The data shown are the means of three independent cultures for each treatment condition and are presented as the fold change of the acetate/synthetic agonist treated samples relative to the respective solvent controls ± SEM; n = 3. The data shown are representative of three independent experiments. n.d: non-detectable. The two tailed Welch's t-test was used to determine the statistical significance of the fold change (between the agonist treatment group and controls) and is annotated as: * < 0.05, ** < 0.005, and *** < 0.0005. **(c)** P38 phosphorylation in human macrophages during 5 mM acetate treatment. **(d)** Western blot of human macrophages treated with 5 mM acetate for 15 min followed by 100 ng/mL LPS challenge.

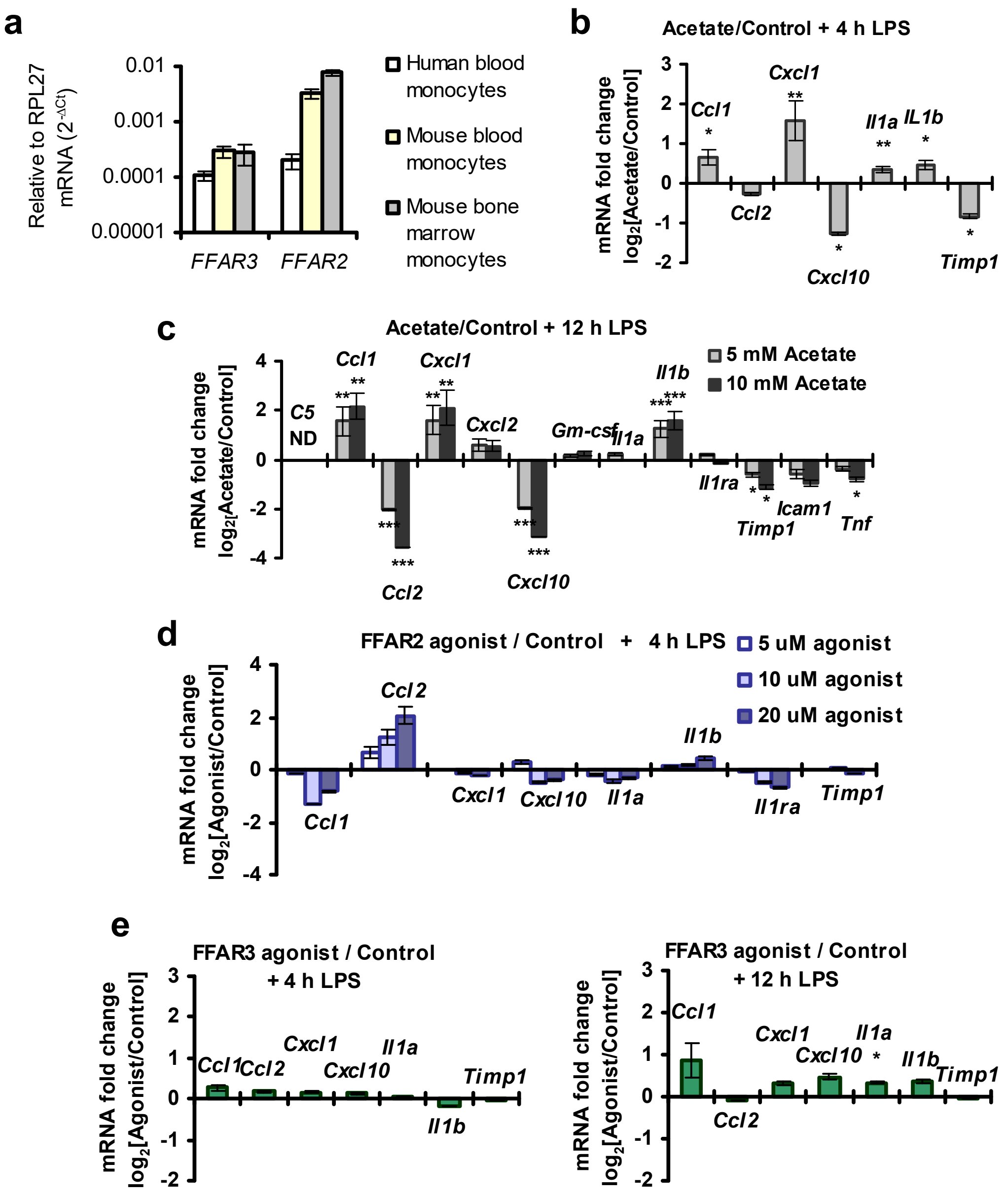

Supplementary Figure 9 | Acetate treatment alters mouse monocyte cytokine expression in a dose-responsive manner that is not reproduced by specific activation of FFAR3 and FFAR2 with synthetic agonists. (a) FFAR2/FFAR3 expression levels in human and mouse monocytes. **(b - e)** Mouse monocytes treated with the indicated concentrations of agonists or solvent controls for 15 min followed by challenge with 100 ng/mL LPS for the indicated times. When not indicated, 5 mM acetate and 10 µM FFAR3 agonist (AR420626) was used, respectively. Cytokine mRNA levels were determined through real-time PCR analysis. The data shown is the mean of three independent cultures for each treatment condition and is presented as the fold change of the acetate or synthetic agonist treated samples relative to the respective solvent controls ± SEM; n = 3. The data shown are representative of three independent experiments. The two tailed Welch's t-test was used to determine the statistical significance of the fold change (between the agonist treatment group and controls) and is annotated as: * < 0.05, ** < 0.005, and *** < 0.0005.

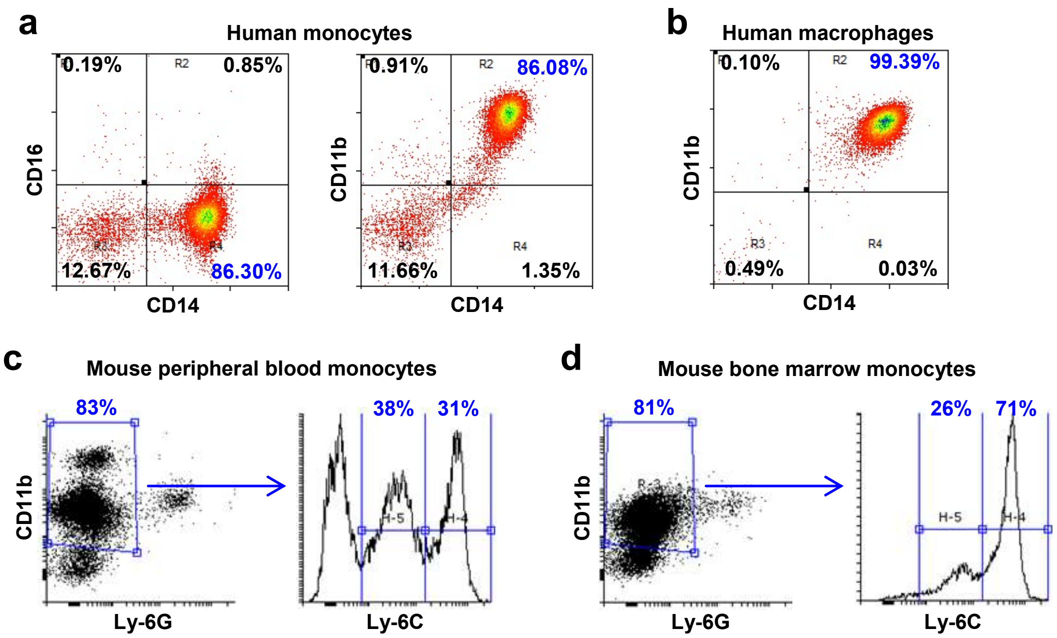

Supplementary Figure 10 | Flow cytometry analysis of moncoyte/macrophage samples. (a) Enriched human peripheral blood monocyte preparations were typically more than 85% CD14^{+}CD16^{-} (Classical). **(b)** Macrophages derived from *in vitro* differentiation of human monocytes were more than 99% CD11b^{+}CD14^{+}. **(c - d)** Mouse monocyte samples were more than 80% CD11b^{+}Ly-6G^{-}. Enriched mouse peripheral blood monocyte preparations contained a mixture of around 38% Ly-6C^{hi} and 31% Ly-6C^{low} subsets, while bone marrow samples contained a higher proportion of around 70% Ly-6C^{hi} monocytes.

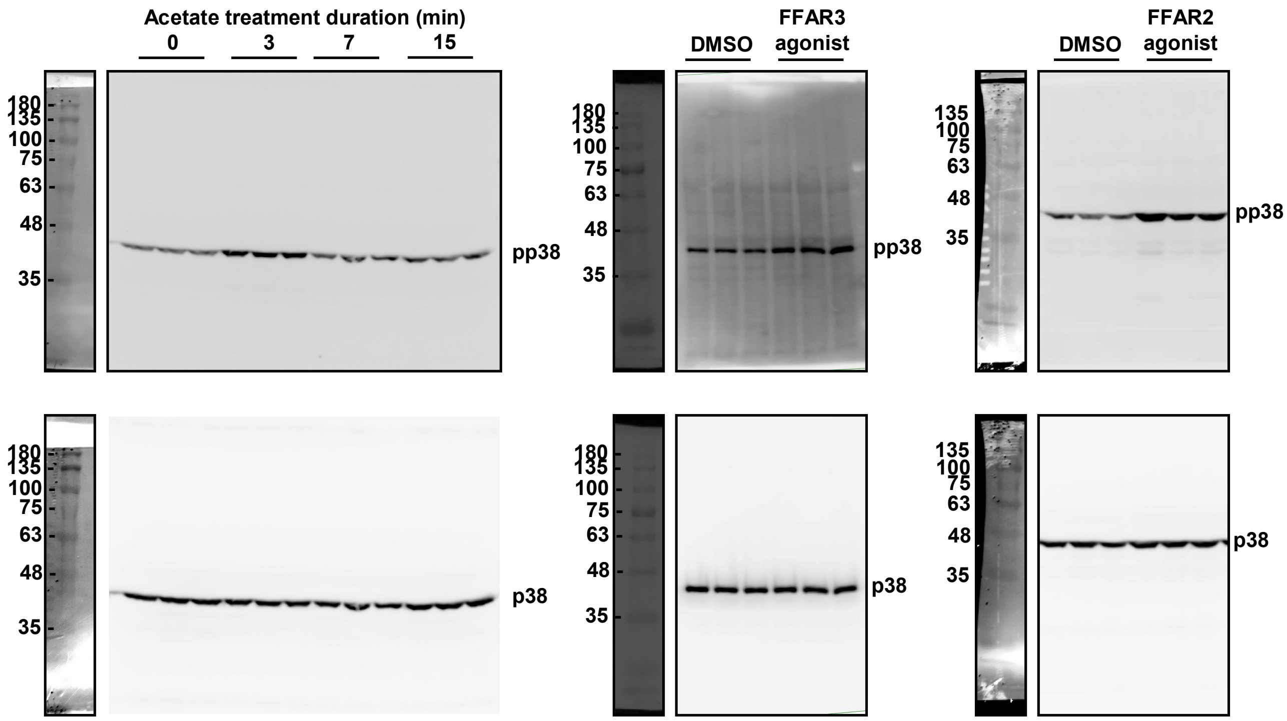

Supplementary Figure 11 | Original full-length anti-p38 western blots used for Figure 4 a.

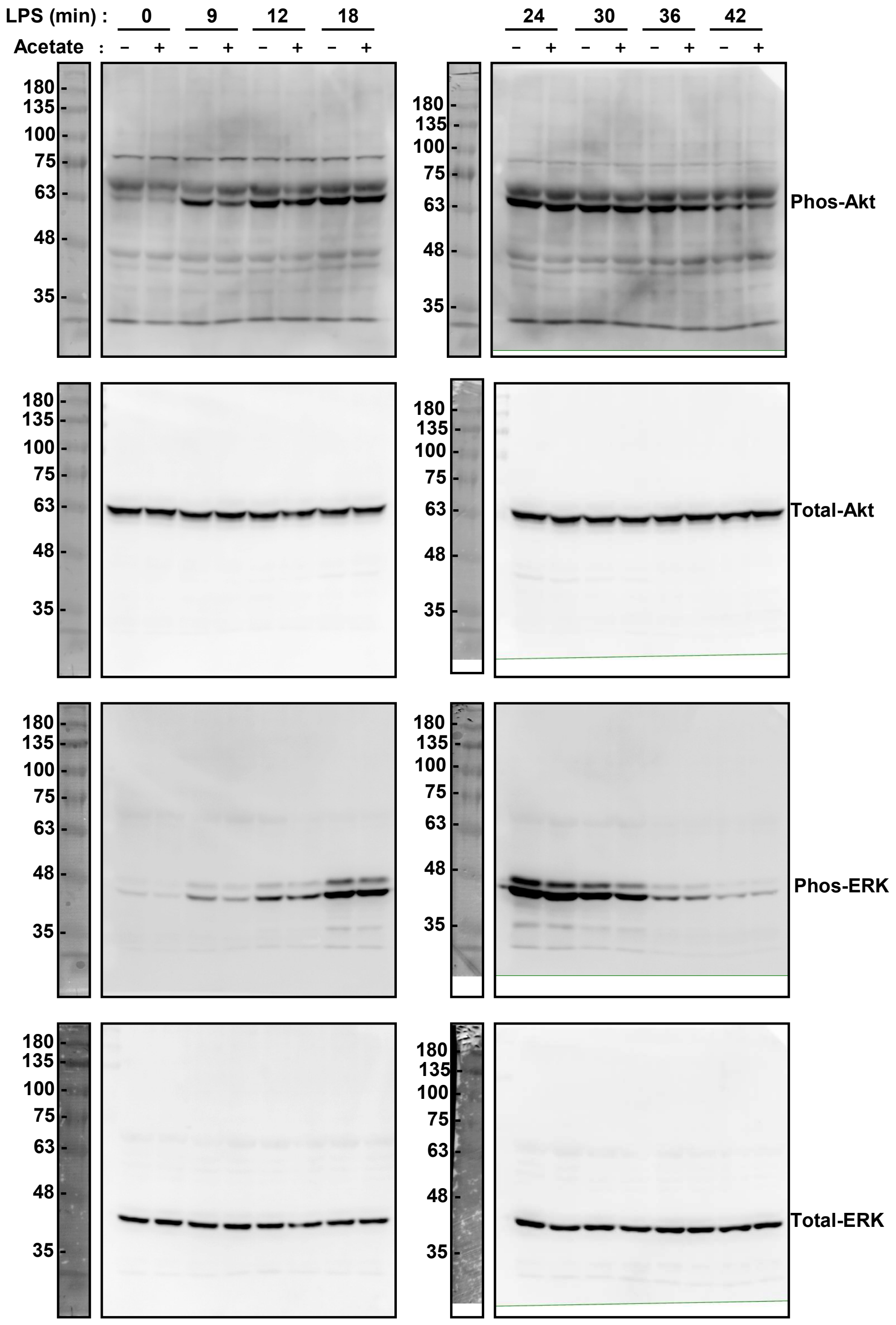

Supplementary Figure 12 | Original full-length anti-Akt and anti-ERK western blots used for Figure 4 c.

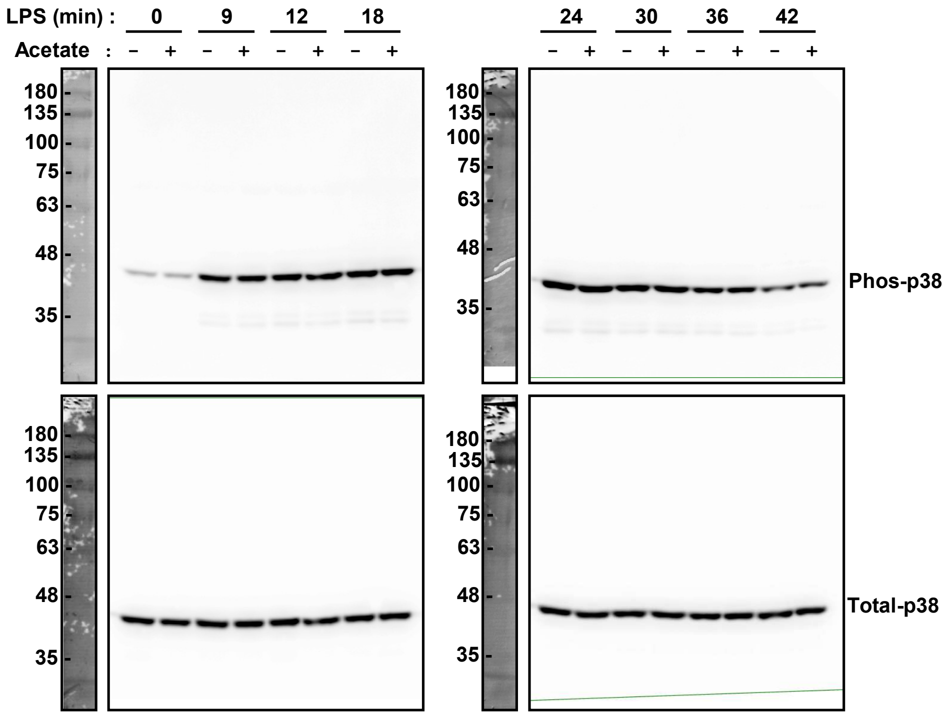

Supplementary Figure 13 | Original full-length anti-p38 western blots used for Figure 4 c.

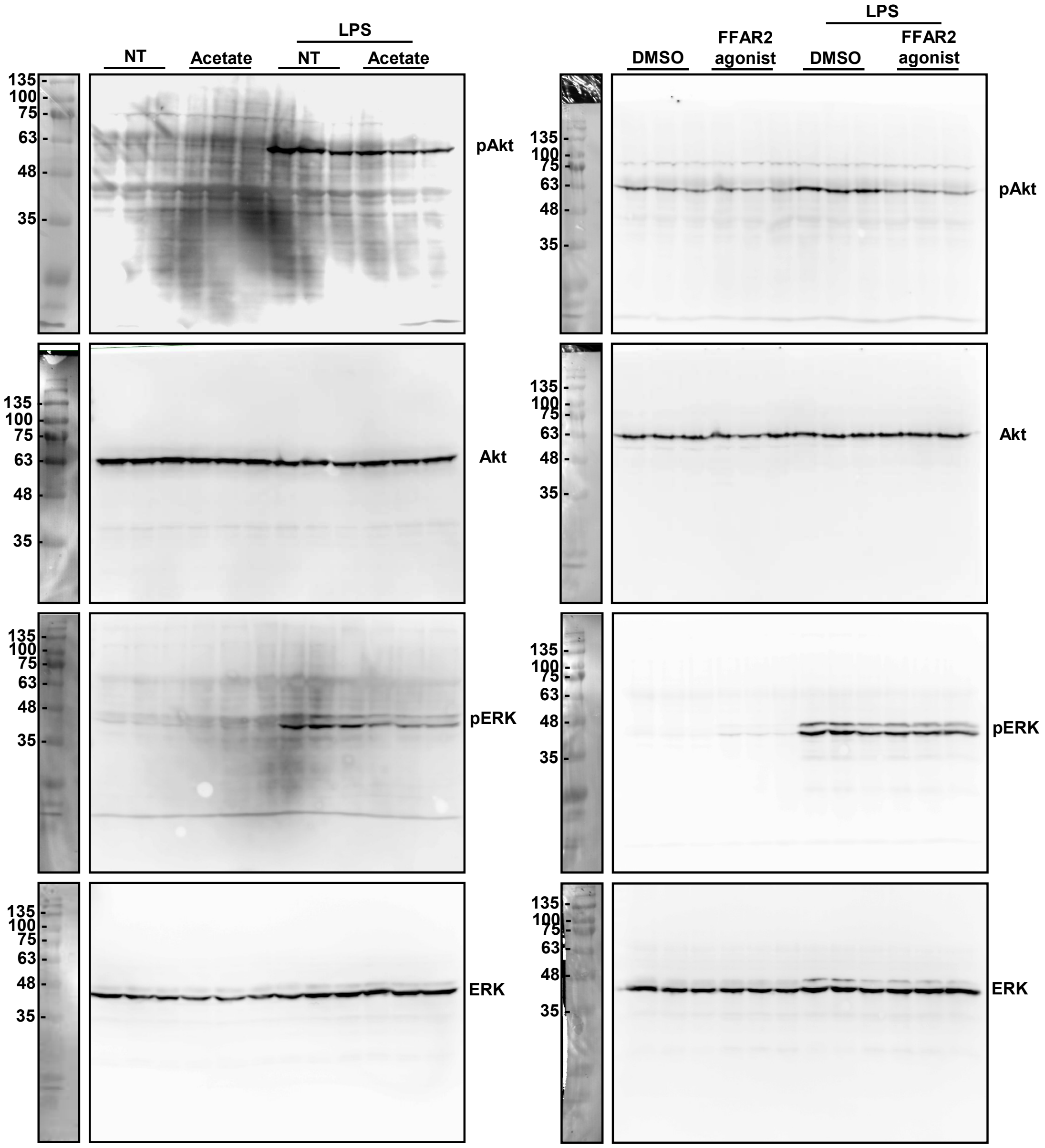

Supplementary Figure 14 | Original full-length anti-Akt western blots used for Figure 4 d.

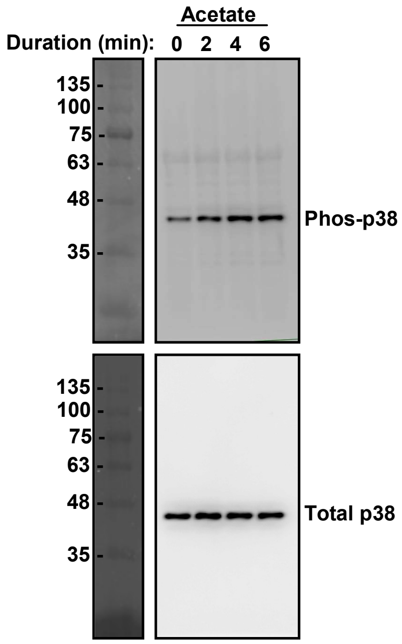

Supplementary Figure 15 | Original full-length anti-p38 western blots used for Figure 5c.

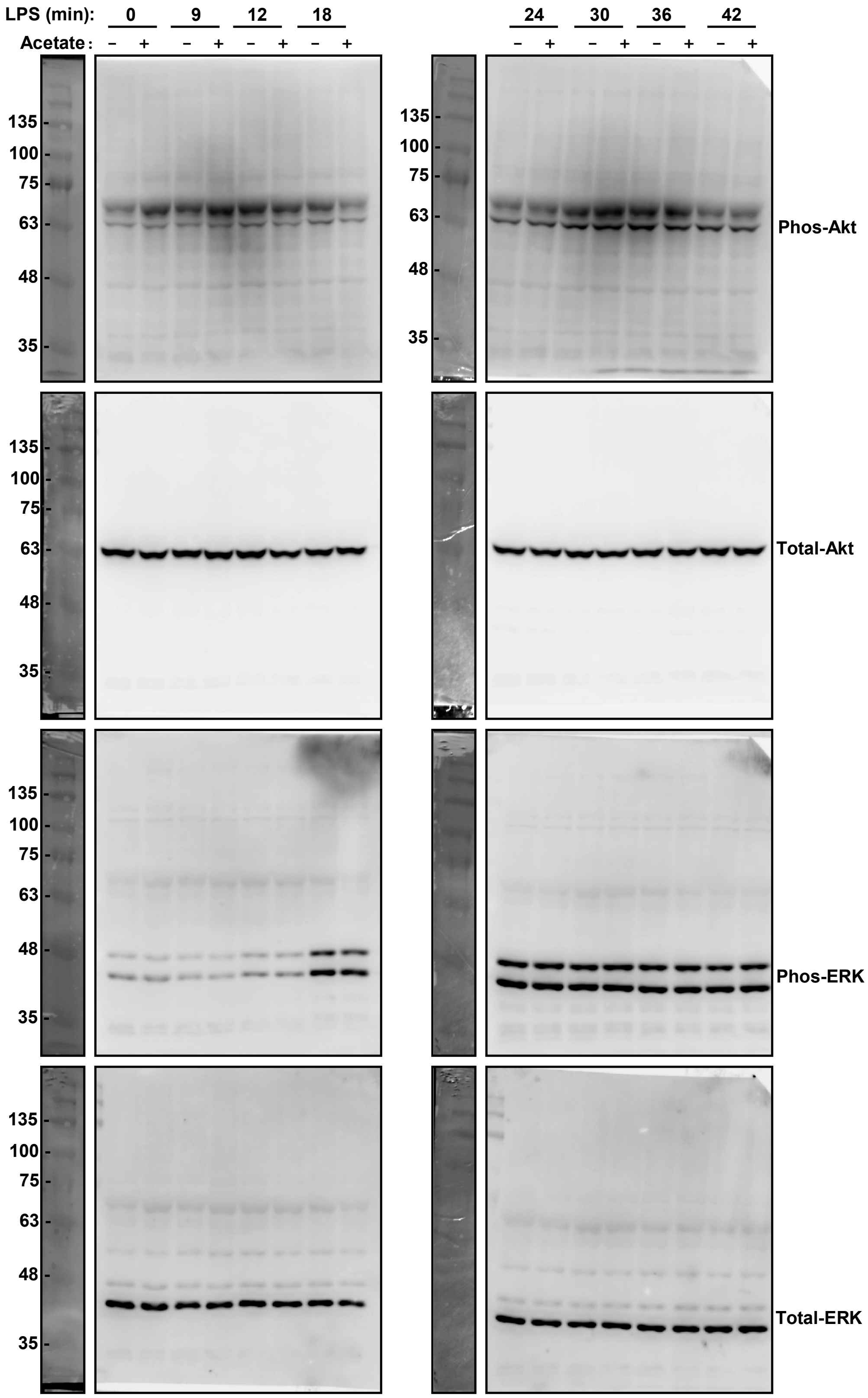

Supplementary Figure 16 | Original full-length anti-Akt and anti-ERK western blots used for Figure 5d.

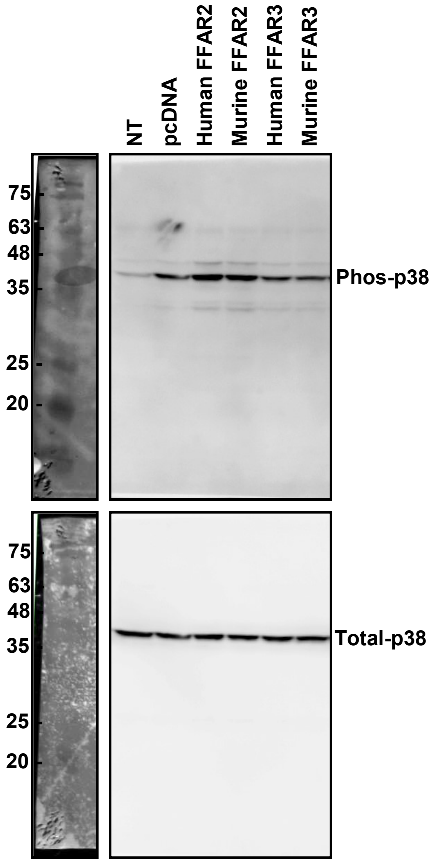

Supplementary Figure 17 | Original full-length anti-p38 western blots used for Figure 6a.
